# Red Algae (Rhodophyta) from the Coast of Madagascar: Preliminary Bioactivity Studies and Isolation of Natural Products

**DOI:** 10.3390/md13074197

**Published:** 2015-07-07

**Authors:** Marie Pascaline Rahelivao, Margit Gruner, Hanta Andriamanantoanina, Bakolinirina Andriamihaja, Ingmar Bauer, Hans-Joachim Knölker

**Affiliations:** 1 Department Chemie, Technische Universität Dresden, Bergstr. 66, 01069 Dresden, Germany; E-Mails: mariepsa@yahoo.fr (M.P.R.); margit.Gruner@tu-dresden.de (M.G.); ingmar.bauer@chemie.tu-dresden.de (I.B.); 2 Centre National de Recherche sur l’Environnement, MESupRes, BP 1739, Antananarivo 101, Madagascar; E-Mail: andriamanantoanina@yahoo.fr; 3 Laboratoire de Chimie Appliquée aux Substances Naturelles, Faculté des Sciences, Université d’Antananarivo, BP 566, Antananarivo 101, Madagascar; E-Mail: b.andriamihaja@moov.mg

**Keywords:** algae, indole alkaloids, steroids, NMR spectroscopy, antimicrobial activity

## Abstract

Several species of red algae (Rhodophyta) from the coastal regions of Madagascar have been investigated for their natural products. The most abundant compound was cholesterol (**5**) in combination with a series of oxidized congeners. The brominated indoles **1**–**3** along with the sesquiterpene debilone (**4**) have been isolated from *Laurencia* *complanata*. For the first time, debilone (**4**) has been obtained from a marine plant. From the methanol extract of *Calloseris* sp., we have achieved the second isolation of the unusual A-ring contracted steroids (−)-2-ethoxycarbonyl-2β-hydroxy-A-nor-cholest-5-en-4-one (**9**) and phorbasterone B (**10**). The crude extracts of *Laurencia* *complanata* exhibited antimicrobial activity against *Bacillus cereus*, *Staphylococcus aureus*, *Streptococcus pneumoniae*, and *Candida albicans*.

## 1. Introduction

Marine organisms may comprise over 80% of the world’s plant and animal species and thus offer an enormous source of novel and potentially biologically active compounds [1]. Among them, algae are one of the richest and most promising sources of bioactive primary and secondary metabolites [2,3]. At present, about 9% of biomedical compounds from marine sources are found in algae [1]. Madagascar, the fourth largest island in the world with a coastline of almost 5000 km, accommodates a wealth of marine organisms in its coastal region. Currently, only few representatives of red algae from Madagascar have been studied for their bioactive compounds. For the red alga *Gelidium* *madagascariense*, Mollion identified an agar polymer in which the methylated fraction contains predominantly 6-*O*-methyl galactose and traces of 4-*O*-methyl galactose [4]. Gerwick and coworkers isolated halogenated monoterpenes from the Madagascan marine red alga *Portieria* *hornemannii* [5]. Two of them proved to be inhibitors of DNA methyl transferase-1 with low-micromolar activity. Recently, the polysaccharide fraction of *Gelidium* sp. was investigated and a gel-forming agar has been identified that is composed of 3,6-anhydro-l-galactopyranose and d-galactopyranose linked via α-(1→4)- and β-(1→3)-glycosidic bonds [6]. The present study aims at the identification of the non-polar natural products of red algae collected in the intertidal zones at the coast of Madagascar.

## 2. Results and Discussion

### 2.1. Laurencia complanata

**Figure 1:**
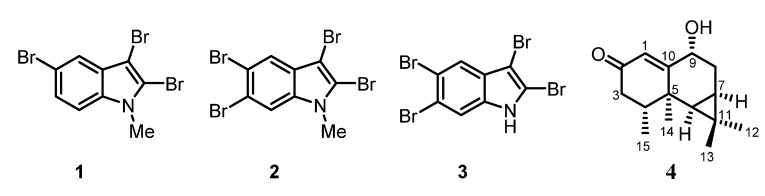
Chemical structures of the bromoindoles **1**–**3** and debilone (**4**).

Dried samples of *Laurencia complanata* were extracted with methanol. Subsequently, the methanol extract was adsorbed at silica gel and subjected to flash chromatography with varying solvents of increasing polarity to provide three fractions. The first fraction, eluting with hexane, gave a mixture of two compounds identified as the brominated indole alkaloids 2,3,5-tribromo-1-methylindole (**1**) and 2,3,5,6-tetrabromo-1-methylindole (**2**) (Figure 1). A ratio of about 3:1 for the two alkaloids was determined by GC-MS and ^1^H NMR spectroscopy. The EI-MS of **1** shows the characteristic pattern for the molecular ions at *m*/*z* = 365, 367, 369, and 371 in the ratio of 1:3:3:1, indicating the presence of three bromine atoms. The molecular formula of **1** was assigned as C_9_H_6_Br_3_N based on EI-MS, ^1^H NMR, and ^13^C NMR data. The EI-MS of compound **2** shows molecular ions at *m*/*z* = 443, 445, 447, 449, and 451 in the ratio of 1:4:6:4:1 indicating the presence of four bromine atoms. The molecular formula C_9_H_5_Br_4_N could be derived from the odd molecular mass and the number of signals in the ^1^H NMR and ^13^C NMR spectra. The spectra of both compounds display a peak for the fragment resulting from the loss of a methyl group. The simple pattern of NMR signals led directly to the assignment of the structures **1** and **2**. The second elution of the methanol extract of *Laurencia complanata* with hexane-ethyl acetate (9:1) afforded a third brominated indole alkaloid: 2,3,5,6-tetrabromoindole (**3**).

Similar to compound **2**, the molecular ions for **3** at *m*/*z* = 429, 431, 433, 435, and 437 in the ratio of 1:4:6:4:1 indicated the presence of four bromine atoms. The molecular formula of **3** was found to be C_8_H_3_Br_4_N based on the GC-MS in combination with ^1^H and ^13^C NMR data. In contrast to **1** and **2**, compound **3** does not show the loss of a methyl group in the mass spectrum. The similarity of the ^1^H and ^13^C NMR spectra of **3** to those of **2** led us to assign compound **3** as 2,3,5,6-tetrabromoindole. Finally, the identity of compounds **1**–**3** was confirmed by the good agreement of their NMR data with those reported in the literature [7,8].

The bromoindoles **1**–**3** were originally isolated by Rinehart and coworkers from *Laurencia* *brongniartii* collected in the Caribbean Sea [7,9]. Subsequently, the same species collected at Komesu, Itoman, Okinawa, Japan in August was also reported as source for **1**–**3** by Higa and Jefford *et al.* [10]. Faulkner *et al.* found the brominated indoles **1** and **2** in the digestive glands of the mollusk *Aplysia* *dactylomela*, probably originating from the algal diet of this animal [11]. Further reports include the isolation of **1** and **3** from *Laurencia* *similis* (Rhodomelaceae) collected at the coast of Hainan island, China [12], and of **1** and **2** from *Laurencia* *decumbens* (Rhodomelaceae) at the coast of Weizhou island, China [13]. In our present work, we describe the first isolation of these natural products from the red alga *Laurencia* *complanata* found at the coast of Madagascar.

After separation of the first two fractions as described above, the crude extract of *Laurencia complanata* was eluted with methanol to provide a third fraction. The aristolane-type sesquiterpene debilone (**4**) was isolated after two further purification steps by column chromatography using a mixture of CH_2_Cl_2_-EtOAc with increasing polarity (Figure 1). The molecular mass of 234 obtained from EI- and ESI-MS in combination with the number and intensity of the ^1^H NMR and ^13^C NMR signals suggested a molecular formula of C_15_H_22_O_2_. The ^1^H NMR spectrum displayed signals for 22 protons, which, according to the DEPT spectrum, include 4 methyl, 2 methylene, and 5 methine groups. In the ^13^C NMR spectrum, signals for 15 carbon atoms, including one carbonyl group at δ = 200.2 ppm (C-2), one oxygenated carbon atom at δ = 72.7 ppm (C-9), and three quaternary carbon atoms at δ = 19.2 (C-11), 37.4 (C-5), and 170.5 ppm (C-10), were identified. Various 2D NMR methods (COSY, HMBC, HSQC, and NOESY) led to the complete assignment of the ^1^H NMR and ^13^C NMR signals and to the elucidation of the relative stereochemistry. Thus, compound **4** was identified as debilone. Comparison of our spectroscopic data with those reported for debilone (**4**) in the literature [14] confirmed the assignment for our compound. Debilone (**4**) was isolated first by Křepinský *et al.* [15] from *Aristolochia debilis* and immediately afterwards by Rücker from *Nardostachys chinensis* [16]. More recently, the tricyclic sesquiterpene has been obtained several times from the same source [14,17,18]. Our present study describes the first isolation of debilone (**4**) from a marine organism.

The crude methanol extract of *Laurencia complanata* was tested for its antimicrobial activity. In an agar diffusion assay at a level of 10 μL of the extract (1 mg/mL) per 6 mm disc, the crude extract produced, after 24 h of growth, inhibition zones of 19.5 mm for *Bacillus cereus*, 20 mm for *Staphylococcus aureus*, 20 mm for *Streptococcus pneumoniae*, and 11.5 mm for *Candida albicans*, which corresponds to a strong inhibiting activity against these microbes (Table 1). A low activity was found against *Klebsiella oxytoca* (7 mm) and *Escherichia coli* (7 mm), whereas no activity was detected against *Enterobacter cloacae* and *Salmonella enteridis*. We showed that at least to some extent the antimicrobial activity can be ascribed to the tetrabromoindole **3** (Table 1), as reported previously by Vairappan and coworkers [19] and Rinehart *et al.* [7]. In addition, Rinehart *et al.* reported for the tetrabromoindole **3** an ID_50_ value of 3.6 μg/mL against L-1210 tumor cells in tissue culture [7]. The brominated indoles **1** and **2** and the methanol fraction containing debilone (**4**) did not contribute significantly to the antimicrobial activity in the agar diffusion test (Table 1).

**Table 1:** Antimicrobial activities of the crude extracts of red algae by agar diffusion test. ^a^

Microbes	Zone of inhibition (Ø in mm) ^b^										
	*Laurencia complanata*				*Grateloupia* sp.	*Gracilaria corticata*	*Halymenia* sp.	*Spyridia* sp.	*Meta-mastophora* sp.	*Calloseris* sp.	*Neurymenia fraxinifolia*
	Crude extract	Mixture of 1 and 2	Compound 3	MeOH fraction (4)							
*Enterobacter cloacae* ATCC 700323	6.5	nt	nt	nt	6	6.5	6	6.5	6	6	6.5
*Klebsiella oxytoca* ATCC 8724	7	nt	nt	nt	6	6.5	6	6.5	6	6	6.5
*Escherichia coli*	7	nt	nt	nt	6	6.5	6	6	6	6	6.5
*Salmonella enteridis*	6	nt	nt	nt	6	6	6	6	6	6	6
*Bacillus cereus* ATCC 13061	19.5	6.5	11	7.5	7	6.5	8	6.5	6.5	6	6.5
*Staphylococcus aureus* ATCC 11632	20	6	12	6	6.5	8	9	6.5	6	6	6.5
*Streptococcus pneumoniae* ATCC 6301	20	6.5	12	6	6	6	12	6	8	6	6
*Candida albicans*	11.5	6.5	8.5	6	6	6	6	6	6	6	6

^a^ Each test was run in triplicate and the mean values are given; ^b^ Concentration of crude methanol extract and isolated compounds: 1 mg/mL, 10 µL solution/6 mm disc; Ø < 7 mm: inactive, 7 mm ≤ Ø < 8 mm: slightly active, 8 mm ≤ Ø < 9 mm: significantly active, Ø ≥ 9 mm: very active; nt = not tested.

### 2.2. Grateloupia sp.

The dried and crushed sample of *Grateloupia* sp. was exhaustively extracted with methanol. The methanol extract was evaporated and the residual solid was extracted with dichloromethane. The dried dichloromethane extract (3.29 g) was subjected to flash chromatography on silica gel and eluted with diethyl ether. The diethyl ether fraction provided 30 mg of cholesterol (**5**) as the major component of *Grateloupia* sp*.* (Halymeniaceae). In the antimicrobial assay, the crude extract of *Grateloupia* sp. showed only minor activity against *Bacillus cereus* (Table 1).

### 2.3. Gracilaria corticata (Gracilariaceae)

**Figure 2:**
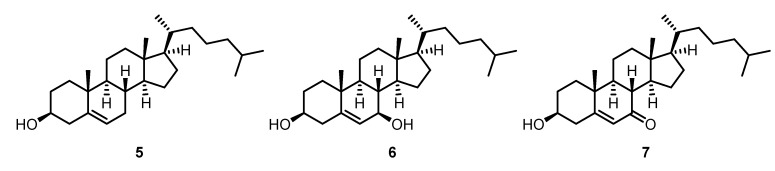
Chemical structures of cholesterol (**5**), cholest-5-ene-3β,7β-diol (**6**), and 3β-hydroxycholest-5-en-7-one (**7**).

After extraction of the dried and crushed sample of *Gracilaria corticata* (Gracilariaceae) with methanol and removal of the solvent, the residual solid was extracted with dichloromethane. The dichloromethane extract was subjected to flash chromatography on silica gel. A first elution with diethyl ether was followed by further elutions with dichloromethane and with ethyl acetate. From the diethyl ether, dichloromethane, and ethyl acetate fractions, we isolated the three known steroids cholesterol (**5**), cholest-5-ene-3β,7β-diol (7β-hydroxycholesterol) (**6**), and 3β-hydroxycholest-5-en-7-one (**7**) (Figure 2). The structures of **5**–**7** have been determined using one- and two-dimensional ^1^H NMR and ^13^C NMR spectroscopy.

The crude extract of *Gracilaria corticata* was found to be only slightly active against *Staphylococcus aureus* (Table 1). No activity was found against *Enterobacter cloacae*, *Klebsiella oxytoca*, *Escherichia coli*, *Salmonella enteridis*, *Bacillus cereus*, *Streptococcus pneumoniae*, and *Candida albicans*.

### 2.4. Halymenia sp. (Halymeniaceae)

After Soxhlet extraction of the dried and crushed sample of *Halymenia* sp. (Halymeniaceae) with diethyl ether, the residue was extracted repeatedly with dichloromethane, ethyl acetate, and methanol. All extracts were subjected to column chromatography on silica gel using a mixture of pentane-diethyl ether in a ratio of 4:1 or 3:2 as eluent. Cholesterol (**5**) was the only component isolable from all fractions, confirming it is the major sterol in most Rhodophyta, as mentioned earlier by Patterson [20].

The methanol extract of *Halymenia* sp. was found to be very active against *Staphylococcus aureus* and *Streptococcus pneumoniae*, but less active against *Bacillus cereus*, and inactive against *Enterobacter cloacae*, *Klebsiella oxytoca*, *Escherichia coli*, *Salmonella enteridis*, and *Candida albicans* (Table 1).

### 2.5. Spyridia sp. (Spyridiaceae)

**Figure 3:**
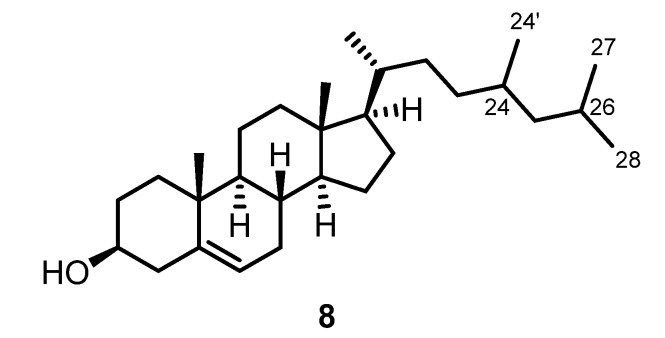
Chemical structure of 24-methyl-25-homocholesterol (**8**).

The plant material of *Spyridia* sp. (Spyridiaceae) was minced, exhaustively extracted with methanol, dried, and then extracted with dichloromethane. Purification of the dichloromethane extract by column chromatography using pentane-diethyl ether (7:3) as eluent led to the isolation of a mixture of cholesterol (**5**) and 24-methyl-25-homocholesterol (**8**) (Figure 3). Compound **8** has been mentioned several times in the literature but to the best of our knowledge only as a hit during a library search from a GC-MS database [21,22,23,24,25]. In the present work, we describe the NMR data of **8** by complete assignment of all ^1^H NMR and ^13^C NMR signals of the mixture with cholesterol (**5**) using COSY, NOESY, HMBC, and HSQC measurements. The configuration of the methyl group at C-24 remains unclear.

The methanol extract of *Spyridia* sp. exhibited no activity in the agar diffusion assay against several microbes, as outlined in Table 1. A moderate activity against the drug-resistant FCM29 strain of *Plasmodium falciparum* with an FCM29 IC_50_ value of 28.53 ± 9.20 µg/mL was observed in the anti-malaria test.

### 2.6. Metamastophora sp. (Corallinaceae)

The methanol extract of *Metamastophora* sp. (Corallinaceae) was evaporated under reduced pressure, leaving a residue that was subjected to flash chromatography on silica gel with various solvents of increasing polarity. The fraction eluting with ethyl acetate was purified by an additional column chromatography on silica gel to give cholest-5-ene-3β,7β-diol (7β-hydroxycholesterol) (**6**) (Figure 2).

The crude extract of *Metamastophora* sp. displayed a low antimicrobial activity against *Streptococcus pneumoniae*, whereas no activity was observed against *Enterobacter cloacae*, *Klebsiella oxytoca*, *Escherichia coli*, *Salmonella enteridis*, *Bacillus cereus*, *Staphylococcus aureus*, and *Candida albicans* (Table 1).

### 2.7. Calloseris sp. (Delesseriaceae)

**Figure 4:**
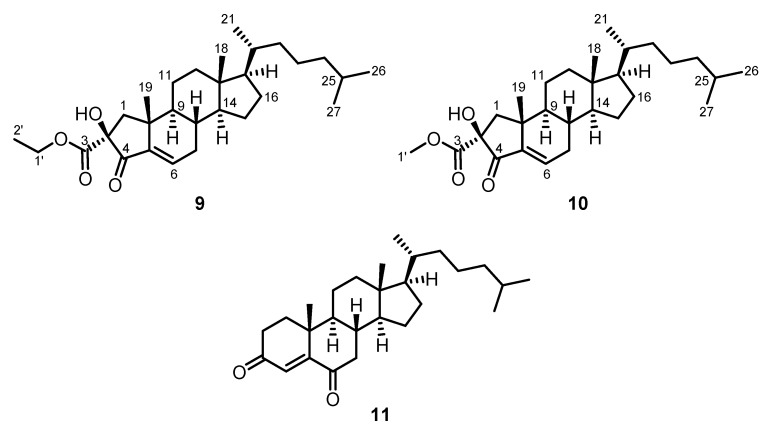
Chemical structures of (−)-2-ethoxycarbonyl-2β-hydroxy-A-nor-cholest-5-en-4-one (**9**), phorbasterone B (**10**), and cholest-4-ene-3,6-dione (**11**).

After extraction of a sample of *Calloseris* sp. (Delesseriaceae) with methanol, the solvent was removed under reduced pressure and the residue was extracted with dichloromethane. The dichloromethane extract was subjected to flash chromatography on silica gel with diethyl ether, dichloromethane, and ethyl acetate. In addition to a mixture of cholesterol (**5**) and 24-methyl-25-homocholesterol (**8**), as isolated from *Spyridia* sp. (see above), the two A-ring contracted steroids, (−)-2-ethoxycarbonyl-2β-hydroxy-A-norcholest-5-en-4-one (**9**) [26] and (−)-2-methoxycarbonyl-2-hydroxy-A-nor-cholest-5-en-4-one [phorbasterone B (**10**)] [27], and cholest-4-ene-3,6-dione (**11**) were isolated from the diethyl ether fraction of *Calloseris* sp. (Figure 4).

Compound **9** was isolated as colorless oil. The molecular mass of 458 was deduced from the ESI-MS by the masses of the protonated molecule and the ammonium adduct (*m*/*z* = 459 [M + H]^+^ and 476 [M + NH_4_]^+^). Infrared absorptions at *ν* = 3440 and 1740 cm^−1^ revealed the presence of hydroxyl and ester groups, respectively. A second absorption for a carbonyl group at 1721 cm^−1^ was assigned to the ketone moiety. The absorption at 1648 cm^−1^ results from a C=C double bond. The ^1^H NMR and ^13^C NMR spectra displayed a close resemblance to cholestane steroids. Obvious differences are the two carbonyl signals at δ = 172.90 (C-3) and 201.05 (C-4) ppm and the signals for an ethoxy group in the ^1^H NMR spectrum at δ = 4.25 ppm (q, *J* = 7.2 Hz, 2 H, H_2_-1′) and δ = 1.27 ppm (t, *J* = 7.2 Hz, 3 H, H_3_-2′). The separated ^1^H NMR signals for the geminal protons at δ = 2.10 ppm (d, *J* = 13.5 Hz, 1 H, H-1b) and 2.18 (d, *J* = 13.6 Hz, 1 H, H-1a) indicated the presence of a contracted ring system. The olefinic proton at δ = 6.74 ppm (t, *J* = 3.5 Hz, 1 H, H-6) displays a downfield shift as compared to cholesterol (**5**), which can be explained by the conjugated electron-withdrawing group. Extensive 2D NMR measurements (COSY, HMBC, HSQC, and NOESY) allowed the assignment of all ^1^H NMR and ^13^C NMR signals for (−)-2-ethoxycarbonyl-2β-hydroxy-A-nor-cholest-5-en-4-one (**9**). With respect to the stereochemistry at C-2, the presence of a NOESY correlation between the hydroxyl proton and the protons of the methyl group (C-19) was in agreement with the β-orientation of the hydroxyl group. Compound **9** has been isolated only once before by Lin and coworkers from the soft coral *Dendronephthya* sp. (Hainan island, China) [26]. Our ^1^H NMR and ^13^C NMR data of **9** were in good agreement with those reported previously (see [26], Table 2, and Section 3.3. Spectroscopic Characterization). The present work on the red alga *Calloseris* sp. constitutes only the second isolation of (−)-2-ethoxycarbonyl-2β-hydroxy-A-nor-cholest-5-en-4-one (**9**).

**Table 2:** ^13^C NMR spectroscopic data of **9** and **10**.^a^

Position	9 from *Calloseris* sp. δ_C_ (150 MHz, CDCl_3_)	9 from *Dendronephthya* sp. [26] δ_C_ (125 MHz, CDCl_3_)	10 from *Calloseris* sp. δ_C_ (150 MHz, CDCl_3_)	10 from *Phorbas amaranthus* [27] δ_C_ (100 MHz, CDCl_3_)
1	46.67	46.7	46.66	nr
2	79.79	79.8	79.88	79.9
3	172.90	172.9	173.35	173.4
4	201.05	201.0	200.89	200.9
5	145.17	145.2	145.09	145.1
6	136.00	135.9	136.26	136.3
7	32.17	32.2	32.19	nr
8	32.32	32.4	32.31	nr
9	49.65	49.7	49.64	nr
10	39.89	39.9	39.91	nr
11	21.78	21.8	21.77	nr
12	39.34	39.5	39.33	nr
13	42.90	42.9	42.89	nr
14	56.25	56.1	56.25	nr
15	24.32	24.3	24.32	nr
16	28.12	28.1	28.12	nr
17	56.09	56.3	56.07	nr
18	12.01	12.0	12.00	nr
19	22.08	22.1	22.07	nr
20	35.73	35.7	35.72	35.7
21	18.73	18.7	18.72	18.7
22	36.15	36.2	36.14	36.1
23	23.83	23.9	23.83	23.8
24	39.47	39.3	39.47	39.5
25	28.00	28.0	28.00	28.0
26	22.81	22.8	22.54	22.5
27	22.55	22.5	22.80	22.8
1′	62.66	62.7	53.37	53.4
2′	14.08	14.2	−	−

^a^ Numbering of the steroidal framework, see Figure 4; nr = not reported.

Compound **10** was obtained as a colorless oil. Its molecular mass of 444 was deduced from the ESI-MS peaks at: *m*/*z* = 445 [M + H]^+^, 462 [M + NH_4_]^+^, and 911 [2M + Na]^+^. The ^1^H NMR and ^13^C NMR spectra of compound **10** proved to be almost identical to those of compound **9** but differ by the presence of signals for a methyl ester instead of those for the ethyl ester, which is matching the difference in the molecular mass of both compounds. Thus, compound **10** could be identified as (−)-2-methoxycarbonyl-2β-hydroxy-A-nor-cholest-5-en-4-one. All ^1^H NMR and ^13^C NMR signals could be unambiguously assigned by 2D NMR measurements (COSY, HMBC, HSQC, and NOESY). Previously, compound **10** was isolated from the sponge *Phorbas amaranthus* by Molinski *et al.* and named phorbasterone B [27]. Our ^1^H NMR and ^13^C NMR data are in full agreement with those reported in the literature ([27], Table 2, and Section 3.3. Spectroscopic Characterization). This report represents only the second isolation of phorbasterone B (**10**). The unusual five-membered A-ring with its characteristic substitution pattern as displayed in the compounds **9** [26], **10**, and other phorbasterones [27] has been described before only for anthosterones A and B, isolated by Andersen and Clardy *et al.* from the sponge *Anthoracuata* *graceae* [28].

Cholest-4-ene-3,6-dione (**11**) was isolated previously from several sources, first from the sponge *Geodia* *cydonium* [29]. Synthetically, it can be easily obtained by oxidation of cholesterol (**5**) with chromium (VI) reagents [30,31]. Our NMR, IR, and UV data of compound **11** were in full agreement with those reported in the literature [29].

The crude extract of *Calloseris* sp. was tested for its antimicrobial activity and found to be inactive against all microbes of our assay (Table 1).

### 2.8. Neurymenia fraxinifolia (Rhodomelaceae)

**Figure 5:**
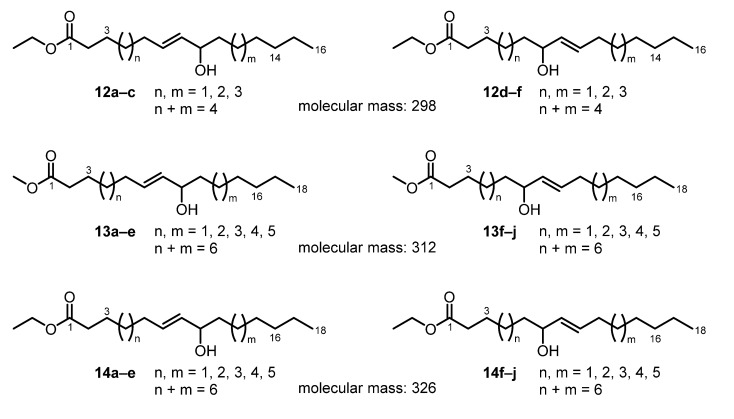
Chemical structures of the allylic alcohols **12**–**14**.

After extraction of the dried and crushed plant material of *Neurymenia fraxinifolia* (Rhodomelaceae) with methanol, the solution was evaporated. The residue was separated by flash chromatography on silica gel using a range of solvents (pentane, diethyl ether, dichloromethane, ethyl acetate, and *n*-butanol). The diethyl ether fraction was further purified by column chromatography on silica gel to afford two fractions. The first fraction provided an amorphous solid, which in the ESI-MS showed signals for at least three components with molecular masses of 298 (*m*/*z* = 281 [M − H_2_O + H]^+^, 316 [M + NH_4_]^+^), 312 (*m*/*z* = 295 [M − H_2_O + H]^+^, 330 [M + NH_4_]^+^), and 326 (*m*/*z* = 309 [M − H_2_O + H]^+^, 344 [M + NH_4_]^+^). In the ^1^H NMR and ^13^C NMR spectra, the mixture appeared almost like a pure substance with the exception of the presence of a methoxy and an ethoxy group in a ratio of about 2:1 that belong to different compounds. Signals are observed for two methyl and three methine groups. Two signals at δ = 5.45 (dd, *J* = 15.4, 7.1 Hz) and 5.59–5.67 (m) showed a shift characteristic of olefinic protons. The ^13^C NMR spectrum displayed signals for one carbonyl group at δ = 174.0 ppm, one oxygenated carbon atom at δ = 73.2 ppm, and two olefinic carbon atoms at δ = 132.3 and 133.2 ppm. The *E*-configuration of the double bond was established by the large coupling constant of *J* = 15.4 Hz between the two olefinic protons and only a weak NOESY correlation between their corresponding signals. Extensive COSY, HSQC, and HMBC experiments led to the assignment for most of the signals of the respective protons and carbon atoms and indicated the presence of an allylic alcohol fragment. However, the position of several of the methylene groups could not be assigned unambiguously to one or the other side of the allylic alcohol moiety within the long alkyl chain. Thus, the position as well as the orientation of the allylic alcohol in the alkyl chain of the fatty acid ester remains unclear. Therefore, several structures appear possible that all would account for the three molecular masses identified in the ESI-MS (Figure 5). The molecular mass of 298 could be represented by the six structures shown for **12**. The molecular mass of 312 leads to 10 possible structures for the methyl ester **13**. Finally, the molecular mass of 326 also provides 10 possible structures for compound **14**.

It is noteworthy that no report in the literature is known for structures **12a–f**. Most probably, compound **12** derives from autoxidation of palmitoleic acid. Thus, only those structures appear reasonable that have the hydroxy group at position 8–10 (**12a–c**, **12f** (*n* = 3, *m* = 1)). Much more information is available in the literature for some of the compounds described by structure **13** (see, for example, [32,33,34,35]). In particular, the compounds **13c** (*n* = 3, *m* = 3), **13d** (*n* = 4, *m* = 2), **13h** (*n* = 3, *m* = 3), and **13i** (*n* = 4, *m* = 2) have been reported several times. They are described as oxidation products of oleic acid formed via hydroperoxide intermediates [36]. No reports are available for the compounds **13f** (*n* = 1, *m* = 5) and **13g** (*n* = 2, *m* = 4), which may be explained by the fact that allylic oxidation of the Δ^9^ double bond in oleic acid does not lead to compounds with a hydroxyl group at position 6 or 7. Examples for the occurrence of structure **13** in natural sources include compounds **13a** and **13i**, which have been isolated from the phytopathogenic fungus *Epichloe* *typhina* [37]. Compared to **13**, the corresponding ethyl esters **14** have been much less investigated. Only reports for the most likely oxidation products **14c** (*n* = 3, *m* = 3) and **14i** (*n* = 4, *m* = 2) are available (see, for example, [38,39,40]). In summary, from the first fraction of *Neurymenia* *fraxinifolia* we have isolated at least three oxidation products of palmitoleic and oleic esters. The precise structures of the isomers could not be determined.

The second fraction of *Neurymenia* *fraxinifolia* was subjected to preparative thin-layer chromatography. The isolated fraction showed only one single spot on the TLC plate, but according to the NMR spectra exhibited a mixture of the three known sterols: 24-methylenecholesterol, cholesterol (**5**), and campesterol.

The methanol extract of *Neurymenia* *fraxinifolia* showed no inhibiting activity against the microbes tested in our assay (Table 1).

## 3. Experimental Section

### 3.1. Plant Material

Samples of the red alga *Laurencia complanata* were collected in May in Italy, located at the southwest coast tip of Madagascar (BOL 169753). Collections were made during the low-tide period, in which the algae are emerging at the surface and can be easily torn from the supporting rocks.

Red algae of the genus *Grateloupia* sp., family Halymeniaceae, were collected in May in Evatraha, a small village north of Fort-Dauphin, located in the southeast of Madagascar (BOL 169751).

Marine algae of the genus *Gracilaria corticata*, family Gracilariaceae, were collected in October in Beravy Tuléar, located on the southwest coast of Madagascar (BOL169754).

Samples of *Halymenia* sp., family Halymeniaceae, were collected in October in Beravy Tuléar on the east coast of Madagascar (BOL 169755).

The red alga *Spyridia* sp., family Spyridiaceae, was collected in February near Fenerive, on the east coast of Madagascar (BOL 169756).

The alga sample of *Metamastophora* sp., family Corallinaceae, was collected in May in Sainte Luce on the southeast coast of Madagascar (BOL 169757).

Samples of the red algae species *Calloseris* sp., family Delesseriaceae, were collected in February in Fenerive at the eastern coastline of Madagascar (BOL 169758).

The sample of the red alga *Neurymenia fraxinifolia*, family Rhodomelaceae, was collected in May in the southeast of Madagascar at Evatraha, a small village north of Fort-Dauphin (BOL 169759).

The red algae have been identified by Dr. Lydiane Mattio and Professor Robert J. Anderson, Biological Sciences Department and Marine Research Institute, University of Cape Town, South Africa. Voucher specimens of the eight species of red algae investigated in this study have been deposited at the Bolus herbarium (BOL) of the University of Cape Town, South Africa. The corresponding BOL accession numbers are given in brackets after the name of the algae.

### 3.2. Extraction and Isolation

The fresh seaweed was washed under tap water, rinsed with distilled water, subsequently dried at 48–50 °C using a universal Binder oven, and then finely powdered in a grinder. In all cases, we extracted the dried and crushed samples of the algae with methanol. Checking the methanol extract by TLC, we obtained different results concerning the polarity of the compounds. Depending on whether the compounds were less polar (best eluted with diethyl ether) or more polar (best eluted with dichloromethane), we performed another extraction with either diethyl ether or dichloromethane, respectively. Following this procedure, we obtained the non-polar compounds of the methanol extract that were purified by column chromatography.

The crushed material of *Laurencia complanata* (15 g) was extracted with methanol at room temperature, the organic extract was evaporated to dryness, and a dark oily residue (methanol extract) was obtained. The methanol extract was subjected to flash chromatography on silica gel using a stepwise gradient of hexane, hexane-ethyl acetate (9:1), and methanol. The fraction eluting with hexane (28 mg) contained a mixture of two alkaloids: 2,3,5-tribromo-1-methylindole (**1**) and 2,3,5,6-tetrabromo-1-methylindole (**2**) in a ratio of 3:1 (GC-MS, NMR). The fraction eluting with hexane-ethyl acetate (113 mg) was further purified by column chromatography on silica gel and provided on elution with pentane-ether (9:1) 97 mg of pure **3**. The fraction eluting with methanol (217 mg) was dissolved in butanol and the butanol extract (123 mg) was subjected to column chromatography on silica gel using dichloromethane-ethyl acetate (5:1) as eluent to get three fractions. The second fraction (13 mg) was further purified by column chromatography on silica gel and afforded on elution with dichloromethane-ethyl acetate (3:2) 1 mg of debilone (**4**).

The dried and crushed *Grateloupia* sp. (400 g) was extracted with methanol. The methanol extract was concentrated to dryness and the residual solid (10 g) was extracted with dichloromethane. Subsequently, the dried dichloromethane extract (3.29 g) was subjected to flash chromatography on silica gel using diethyl ether as eluent. The diethyl ether fraction was further subjected to column chromatography on silica gel and provided on elution with pentane-diethyl ether (4:1) 30 mg of cholesterol (**5**).

For the methanol extract of *Gracilaria corticata*, the algae powder (600 g) was added to methanol and suspended with a homogenizer. After filtration, the methanol was removed from the combined extracts by evaporation under reduced pressure, and the residue (15 g) was extracted with dichloromethane. The dichloromethane extract (3 g) was subjected to flash chromatography on silica gel. The column was eluted with diethyl ether, dichloromethane, and then with ethyl acetate. From the diethyl ether, dichloromethane, and ethyl acetate fractions, 60 mg of cholesterol (**5**), 5 mg of 3β-hydroxycholest-5-en-7-one (**7**), and 4.5 mg of cholest-5-ene-3β,7β-diol (7β-hydroxycholesterol) (**6**) were isolated.

The crushed *Halymenia* sp. (38 g) was extracted with four different solvents of increasing polarity, namely diethyl ether, dichloromethane, ethyl acetate, and methanol. The diethyl ether extract (180 mg) was purified by column chromatography on silica gel and afforded on elution with pentane-diethyl ether (4:1) 6 mg of cholesterol (**5**). The dichloromethane (63 mg) and the ethyl acetate (90 mg) extracts were treated in the same way to obtain 2.3 mg and 3 mg of **5**. Compound **5** (1 mg) was also obtained by column chromatography of the methanol extract on silica gel and elution with pentane-diethyl ether (3:2).

Crushed *Spyridia* sp. (2 kg) was extracted with methanol and the insoluble materials were removed by filtration. Evaporation of the methanol from the soluble extract gave a dark oily residue. The methanol extract (42 g) was extracted with dichloromethane at room temperature. Subsequently, the dichloromethane extract (7 g) was further separated by two column chromatographies on silica gel with pentane-diethyl ether (7:3) as eluent to afford 387 mg of a mixture (one TLC spot) of cholesterol (**5**) and 24-methyl-25-homocholesterol (**8**). A ratio of about 5:4 for the two steroids was determined by GC-MS and ^1^H NMR.

The crushed material of *Metamastophora* sp. (300 g) was extracted with methanol and the insoluble components were removed by filtration. Evaporation of the methanol from the extract gave 6 g of a dark oily residue. The crude extract was subjected to flash chromatography on silica gel and was eluted with different solvents of increasing polarity, namely pentane, diethyl ether, dichloromethane, ethyl acetate, and methanol. The ethyl acetate extract (52 mg) was fractionated by column chromatography on silica gel using ethyl acetate as eluent to afford 3 mg of cholest-5-ene-3β,7β-diol (7β-hydroxycholesterol) (**6**).

The crushed material of *Calloseris* sp. (550 g) was extracted with methanol. The extract was filtered, evaporated, and the residual solid (13 g) was extracted with dichloromethane. The dichloromethane extract (4.6 g) was subjected to flash chromatography on silica gel and eluted first with pentane to remove the non-polar fractions and then with diethyl ether. The diethyl ether fraction (2.9 g) was subjected to column chromatography on silica gel and provided on elution with pentane-diethyl ether (4:1) three fractions. Fraction 1 (100 mg) was further purified by column chromatography on silica gel using pentane-diethyl ether (9:1) as eluent and afforded 25 mg of (−)-2-ethoxycarbonyl-2β-hydroxy-A-nor-cholest-5-en-4-one (**9**). Fraction 2 (200 mg) was also further purified by column chromatography on silica gel using pentane-diethyl ether (4:1) as eluent to provide fraction 2-1 and fraction 2-2. Fraction 2-1 gave 45 mg of phorbasterone B (**10**) and fraction 2-2 gave 39 mg of cholest-4-ene-3,6-dione (**11**). Fraction 3 (710 mg) showed only one single spot on TLC analysis but contained a mixture of cholesterol (**5**) and 24-methyl-25-homocholesterol (**8**).

The crushed material of *Neurymenia fraxinifolia* (500 g) was extracted with methanol. The methanol extract (11 g) was subjected to flash chromatography on silica gel using different solvents of increasing polarity (pentane, diethyl ether, dichloromethane, ethyl acetate, and *n*-butanol). The diethyl ether fraction was subjected to column chromatography on silica gel and elution with pentane-diethyl ether (4:1) afforded two fractions. The first fraction provided 2 mg of a mixture of the compounds **12**–**14**. The second fraction (37 mg) was subjected to a further column chromatography on silica gel with pentane-diethyl ether (4:1) followed by a preparative TLC using pentane-diethyl ether (1:1). The product (6 mg) appeared as one spot on TLC analysis but contained a mixture of 24-methylenecholesterol, cholesterol (**5**), and campesterol in a ratio of about 3:2:1 according to GC-MS and the ^1^H NMR spectrum.

### 3.3. Spectroscopic Characterization

General: Optical rotations were determined on a Perkin Elmer 341 polarimeter at a wavelength of 589 nm (sodium D line) using a 1.0-decimeter cell with a total volume of 1.0 mL. UV spectra were measured on a Perkin Elmer Lambda 25 UV-Vis spectrometer. Fluorescence spectra were measured on a Varian Cary Eclipse. IR spectra were recorded on a Thermo Nicolet Avatar 360 E.S.P. FT-IR spectrometer using the ATR technique (attenuated total reflection). NMR spectra were recorded on Bruker AC 300-P and AVANCE III 600 spectrometers. The chemical shifts δ are reported in ppm using the non-deuterated solvent as internal standard. Assignment of the ^1^H NMR and ^13^C NMR signals was achieved using the 2D NMR methods COSY, HSQC, HMBC, and NOESY. The mass spectra were measured by GC-MS coupling with an Agilent Technologies 6890N GC system equipped with a 5973N Mass Selective Detector (electron impact, 70 eV). ESI-MS were recorded on a Bruker-Esquire mass spectrometer with an ion trap detector; positive and negative ions were detected. Thin layer chromatography was performed on aluminum plates coated with silica gel 60-F_254_ (Merck). Preparative TLC was carried out with glass plates (20×20 cm, Merck) coated with a 0.25 mm layer of silica gel (60-F_254_). For visualization, the plates were analyzed under UV light or treated with a solution of 0.5 g vanillin dissolved in 100 mL of 80/20 (v/v) sulfuric acid/ethanol and subsequently heated.

2,3,5-Tribromo-1-methylindole (**1**) and 2,3,5,6-tetrabromo-1-methylindole (**2**), 3:1 mixture: amorphous solid.

2,3,5-Tribromo-1-methylindole (**1**) (NMR data from the 3:1 mixture of **1** and **2**): ^1^H NMR (300 MHz, CDCl_3_): δ (ppm) = 3.80 (s, 3 H, CH_3_), 7.17 (d, *J* = 8.7 Hz, 1 H, H-7), 7.34 (dd, *J* = 8.7 Hz, 1.8 Hz, 1 H, H-6), 7.66 (d, *J* = 1.8 Hz, 1 H, H-4); ^13^C NMR and DEPT (125 MHz, CDCl_3_): δ (ppm) = 32.52 (CH_3_), 91.93 (C-3), 111.10 (C-7), 114.16 (C-5), 116.25 (C-2), 121.39 (C-4), 125.79 (C-6), 128.37 (C-3a), 134.99 (C-7a); GC-MS (EI, 70 eV): *m*/*z* (%) = 371/369/367/365 (31/96/100/35, M^+^), 356/354/352/350 (3/9/9/3), 288 (7), 249/247/245 (2/5/2), 209/207 (8/9), 194/192 (11/11), 128 (15), 87 (9).

2,3,5,6-Tetrabromo-1-methylindole (**2**) [7,41,42] (NMR data from the 3:1 mixture of **1** and **2**): ^1^H NMR (300 MHz, CDCl_3_): δ (ppm) = 3.77 (s, 3 H, CH_3_), 7.60 (s, 1 H, H-7), 7.78 (s, 1 H, H-4); ^13^C NMR and DEPT (125 MHz, CDCl_3_): δ ppm = 32.66 (CH_3_), 91.98 (C-3), 114.48 (C-7), 116.39 (C-5), 117.23 (C-2), 118.44 (C-6), 123.13 (C-4), 127.49 (C-3a), 135.77 (C-7a); GC-MS (EI, 70 eV): *m*/*z* (%) = 451/449/447/445/443 (15/60/100/66/17, M^+^), 434/432/430 (7/12/7), 368 (7), 366 (7), 289/287/285 (9/19/10), 274/272/270 (6/11/5), 223 (7), 208/206 (5/5), 167/165 (5/5), 127 (7), 112 (11), 86 (7).

2,3,5,6-Tetrabromoindole (**3**): Amorphous solid; mp = 151–152 °C (decomposition) (lit. 153–154 °C [8]); UV (MeOH): λ (nm) = 234, 297, 305 (sh); fluorescence (MeOH): λ_max_ (297 nm) = 317 nm; IR (ATR): *ν* (cm^−1^) = 3382, 2921, 1608, 1555, 1502, 1431, 1365, 1303, 1226, 1086, 995, 862; ^1^H NMR (600 MHz, CDCl_3_): δ (ppm) = 7.61 (s, 1 H, H-7), 7.77 (s, 1 H, H-4), 8.30 (br s, 1 H, NH); ^13^C NMR and DEPT (150 MHz, CDCl_3_): δ (ppm) = 93.75 (C-3), 112.15 (C-2), 115.39 (C-7), 116.69 (C-5), 119.00 (C-6), 123.18 (C-4), 128.22 (C-3a), 134.93 (C-7a); GC-MS (EI, 70 eV): *m*/*z* (%) = 437/435/433/431/429 (16/66/100/70/18, M^+^), 356/354/352/350 (8/26/26/8), 275/273/271 (13/26/13), 216 (9), 194/192 (9/10), 167/165 (7/8), 136 (13), 112 (8), 86 (14); ESI-MS (−10 V): *m*/*z* = 428/430/432/434/436 [M − H]^−^; Anal. calcd for C_8_H_3_Br_4_N: C 22.20, H 0.70, N 3.24; found: C 23.10, H 0.62, N 3.22%.

(1a*R*,3*R*,7*R*,7a*R*,7b*S*)-3-Hydroxy-1,1,7,7a-tetramethyl-1,1a,2,3,6,7,7a,7b-octahydro-5*H*-cyclopropa[*a*]naphthalen-5-one (9-hydroxyaristol-1(10)-en-2-one, debilone) (**4**) [14,43,44]: Colorless needles; UV (MeOH): λ (nm) = 218; fluorescence (MeOH): λ_max_ (218 nm) = 291 nm; ^1^H NMR (600 MHz, CDCl_3_): δ (ppm) = 0.77 (d, *J* = 9.0 Hz, 1 H, H-6), 0.93 (s, 3 H, H_3_-13), 0.95–0.97 (m, 1 H, H-7), 1.08 (d, *J* = 6.4 Hz, 3 H, H_3_-15), 1.09 (s, 3 H, H_3_-12), 1.46 (s, 3 H, H_3_-14), 1.76 (dt, *J* = 16.0, 3.9 Hz, 1 H, H-8b), 2.27–2.39 (m, 4 H, H-8a, H-4, H_2_-3), 4.23 (br s, 1 H, H-9), 5.82 (s, 1 H, H-1); ^13^C NMR derived from HSQC and HMBC (150 MHz, CDCl_3_): δ (ppm) = 15.0 (C-15), 16.4 (C-7), 17.5 (C-13), 19.2 (C-11), 23.9 (C-14), 28.0 (C-8), 29.2 (C-12), 31.9 (C-6), 37.1 (C-4), 37.4 (C-5), 42.9 (C-3), 72.7 (C-9), 126.5 (C-1), 170.5 (C-10), 200.2 (C-2); GC-MS (EI, 70 eV): *m*/*z* (%) = 234 (8, M^+^), 216 (61), 201 (54), 192 (72), 173 (61), 159 (89), 145 (80), 131 (62), 121 (54), 105 (87), 91 (89), 77 (62), 69 (51), 55 (60), 41 (100); ESI-MS (10 V): *m/z* = 235 [M + H]^+^, 469 [2M + H]^+^.

24-Methyl-25-homocholesterol (**8**): Colorless solid; ^1^H NMR (600 MHz, CDCl_3_): δ (ppm) = 0.69 (s, 3 H, H_3_-18), 0.82 (d, *J* = 6.8 Hz, 3 H, H_3_-27), 0.84 (d, *J* = 6.8 Hz, 3 H, H_3_-28), 0.87 (d, *J* = 7.5 Hz, 3 H, H_3_-24′), 0.92–0.95 (m, 2 H, H-9, H-24), 0.93 (d, *J* = 6.4 Hz, 3 H, H_3_-21), 0.96–1.00 (m, 1 H, H-22b), 1.00–1.02 (m, 1 H, H-14), 1.02 (s, 3 H, H_3_-19), 1.03–1.20 (m, 6 H, H-1b, H12b, H-15b, H-17, H-23b, H-25b), 1.24–1.40 (m, 5 H, H-16b, H-20, H-22a, H-23a, H-25a), 1.44–1.62 (m, 6 H, H-2b, H-7b, H-8, H_2_-11, H-15a), 1.63–1.72 (m, 1 H, H-26), 1.80–1.89 (m, 3 H, H-1a, H-2a, H-16a), 1.95–1.99 (m, 1 H, H-7a), 1.99–2.04 (m, 1 H, H-12a), 2.23–2.27 (m, 1 H, H-4b), 2.28–2.33 (m, 1 H, H-4a), 3.50–3.57 (m, 1 H, H-3), 5.36 (br d, *J* = 5.3 Hz, 1 H, H-6); ^13^C NMR and DEPT (150 MHz, CDCl_3_): δ (ppm) = 11.84 (C-18), 12.31 (C-24′), 18.81 (C-21), 18.95 (C-27), 19.39 (C-28), 19.58 (C-19), 21.07 (C-11), 22.99 (C-23), 24.30 (C-15), 26.34 (C-25), 28.23 (C-16), 28.91 (C-26), 31.66 (C-2), 31.89 (C-7, C-8), 33.89 (C-22), 36.26 (C-20), 36.49 (C-10), 37.24 (C-1), 39.76 (C-12), 42.31 (C-4), 42.59 (C-13), 46.04 (C-24), 50.11 (C-9), 56.01 (C-17), 56.75 (C-14), 71.81 (C-3), 121.72 (C-6), 140.75 (C-5); GC-MS (EI, 70 eV): *m*/*z* (%) = 414 (100, M^+^), 399 (35), 396 (52), 381 (34), 329 (51), 303 (48), 273 (22), 255 (29), 213 (40), 145 (41), 107 (38), 105 (40), 55 (44), 43 (67).

(−)-2-Ethoxycarbonyl-2β-hydroxy-A-nor-cholest-5-en-4-one (**9**): Colorless oil; ${\left[\text{α}\right]}_{\text{D}}^{20}$ = −12.7 (c = 0.11, MeOH) (lit. −20.8 (c = 0.8, CHCl_3_) [26]); CD Δ*ε* (MeOH): λ (nm): +0.42 (239), +0.25 (269); UV (MeOH): λ (nm) = 246; fluorescence (MeOH): λ_max_ (246 nm) = 298 nm; IR (ATR): *ν* (cm^−1^) = 3441 (br), 2927, 2867, 2846, 1740, 1721, 1648, 1461, 1375, 1264, 1230, 1179, 1101, 1056, 894; ^1^H NMR (600 MHz, CDCl_3_): δ (ppm) = 0.74 (s, 3 H, H_3_-18), 0.87 (d, *J* = 6.8 Hz, 3 H, H_3_-26), 0.88 (d, *J* = 6.4 Hz, 3 H, H_3_-27), 0.94 (d, *J* = 6.4 Hz, 3 H, H_3_-21), 1.02–1.06 (m, 2 H, H-9, H-22b), 1.07–1.18 (m, 6 H, H-14, H-15b, H-17, H-23b, H_2_-24), 1.21 (s, 3 H, H_3_-19), 1.20–1.25 (m, 1 H, H-12b), 1.27 (t, *J* = 7.2 Hz, 3 H, H_3_-2′), 1.29–1.40 (m, 4 H, H-16b, H-20, H-22a, H-23a), 1.47–1.59 (m, 3 H, H_2_-11, H-25), 1.59–1.66 (m, 1 H, H-15a), 1.67–1.72 (m, 1 H, H-8), 1.82–1.92 (m, 2 H, H-7b, H-16a), 2.05–2.10 (m, 1 H, H-12a), 2.10 (d, *J* = 13.5 Hz, 1 H, H-1b), 2.18 (d, *J* = 13.6 Hz, 1 H, H-1a), 2.40 (dt, *J* = 21.8, 4.5 Hz, 1 H, H-7a), 3.84 (s, 1 H, OH), 4.25 (q, *J* = 7.2 Hz, 2 H, H_2_-1′), 6.74 (t, *J* = 3.6 Hz, 1 H, H-6); ^13^C NMR and DEPT (150 MHz, CDCl_3_): see Table 2; ESI-MS (25 V): *m*/*z* = 459 [M + H]^+^, 476 [M + NH_4_]^+^.

Phorbasterone B (**10**): Colorless oil; CD Δ*ε* MeOH (λ nm): −0.24 (239), +0.29 (269); UV (MeOH): λ (nm) = 246; fluorescence (MeOH): λ_max_ (246 nm) = 298 nm; IR (ATR): *ν* (cm^−1^) = 3456 (br), 2924, 2852, 1743, 1718, 1649, 1459, 1376, 1267, 1232, 1175, 1102, 1054, 960, 886, 767, 716; ^1^H NMR (600 MHz, CDCl_3_): δ (ppm) = 0.73 (s, 3 H, H_3_-18), 0.87 (d, *J* = 6.4 Hz, 3 H, H_3_-27), 0.88 (d, *J* = 6.8 Hz, 3 H, H_3_-26), 0.94 (d, *J* = 6.8 Hz, 3 H, H_3_-21), 0.99–1.12 (m, 3 H, H-9, H-14, H-22b), 1.12–1.20 (m, 5 H, H-15b, H-17, H-23b, H_2_-24), 1.19–1.24 (m, 1 H, H-12b), 1.21 (s, 3 H, H_3_-19), 1.32–1.42 (m, 4 H, H-16b, H-20, H-22a, H-23a), 1.48–1.55 (m, 2 H, H-11b, H-25), 1.58–1.64 (m, 2 H, H-11a, H-15a), 1.66–1.74 (m, 1 H, H-8), 1.82–1.92 (m, 2 H, H-7b, H-16a), 2.07 (dt, *J* = 12.4, 3.4 Hz, 1 H, H-12a), 2.11 (d, *J* = 13.5 Hz, 1 H, H-1b), 2.19 (d, *J* = 13.9 Hz, 1 H, H-1a), 2.41 (dt, *J* = 21.5, 4.9 Hz, 1 H, H-7a), 3.79 (s, 3 H, H_3_-1′), 3.80 (s, 1 H, OH), 6.75 (t, *J* = 3.6 Hz, 1 H, H-6); ^13^C NMR and DEPT (150 MHz, CDCl_3_): see Table 2; ESI-MS (25 V): *m/z* = 445 [M + H] ^+^, 462 [M + NH_4_] ^+^, 911 [2M + Na] ^+^.

Mixture of allylic alcohols **12**–**14**: Amorphous solid; ^1^H NMR (600 MHz, CDCl_3_): δ (ppm) = 0.83–0.93 (m, 3 H), 1.23–1.35 (m, about 19 H), 1.43–1.58 (m, 2 H), 1.59–1.66 (m, 2 H), 2.03 (q, *J* = 7.4 Hz, 2 H), 2.26–2.33 (m, 2 H), 3.68 (s, 3 H, OCH_3_), 4.04 (br q, *J* = 6.4 Hz, 1 H, OCH), 4.13 (q, *J* = 7.1 Hz, 2 H, OCH_2_), 5.45 (dd, *J* = 15.4, 7.1 Hz, 1 H), 5.59–5.67 (m, 1 H); ^13^C NMR and DEPT (signals partially derived from HSQC and HMBC) (150 MHz, CDCl_3_): δ (ppm) = 14.1 (CH_3_), 14.3 (CH_3_), 22.6 (CH_2_), 24.9 (CH_2_), 25.4 (CH_2_), 28.7–29.7 (several CH_2_), 31.8 (CH_2_), 32.1 (CH_2_), 34.4 (CH_2_), 37.3 (CH_2_), 60.2 (CH_2_), 73.2 (CH), 132.3 (CH), 133.2 (CH), 174.0 (C); ESI-MS (10 V): *m*/*z* = 281 [M – H_2_O + H]^+^ (**12**), 295 [M − H_2_O + H]^+^ (**13**), 309 [M − H_2_O + H]^+^ (**14**), 316 [M + NH_4_]^+^ (**12**), 330 [M + NH_4_]^+^ (**13**), 344 [M + NH_4_]^+^ (**14**), 561 [2M − 2H2O + H]^+^ (**12** + **12**), 575 [2M − 2H2O + H]^+^ (**12** + **13**), 589 [2M − 2H2O + H]^+^ (**13** + **13**) (**12** + **14**), 603 [2M − 2H2O + H]^+^ (**13** + **14**), 617 [2M − 2H2O + H]^+^ (**14** + **14**).

### 3.4. Biological Testing

Antimicrobial assay: The antimicrobial activities were determined using the agar diffusion technique in Petri dishes. The crude algal extracts and the three compounds **1**, **2**, and **3** were tested for their antimicrobial activity against four Gram-negative bacteria: *Enterobacter cloacae* (ATCC 700323), *Klebsiella oxytoca* (ATCC 8724), *Escherichia coli*, *Salmonella enteridis*; three Gram-positive bacteria: *Bacillus cereus* (ATCC 13061), *Staphylococcus aureus* (ATCC 11632), *Streptococcus pneumoniae* (ATCC 6301); and against one yeast strain: *Candida albicans*. The pathogens were supplied by the Laboratoire de Microbiologie de l’Environnement (LME), Centre National de Recherche sur l’Environnement (CNRE), Antananarivo, Madagascar. The crude extracts and the compounds **1**–**3** were dissolved in methanol at a concentration of 1 mg/mL. A sample (10 μL) of each solution was added via a pipette onto a sterile antibiotic filter disc of 6 mm diameter and oven dried at 40–50 °C. The discs were placed on Müller-Hinton agar plates that had been inoculated with the microorganisms mentioned above. The plates were incubated for 24 h at 37 °C for the bacteria and for 48 h at 25 °C for the yeast. The diameters of the inhibition zones generated around the discs were measured (Ø in mm). The tests were performed in triplicate and the mean values are given in Table 1. Methanol, used to dissolve the extracts and the compounds, was checked for the absence of antimicrobial activity. The diameters of the halos of inhibition can be rationalized on a qualitative basis as follows: Ø < 7 mm: inactive, 7 mm ≤ Ø < 8 mm: slightly active, 8 mm ≤ Ø < 9 mm: significantly active, Ø ≥ 9 mm: very active.

Antimalaria test: The antiplasmodial activity against the FCM29 strain of *Plasmodium falciparum* was determined by using the microfluorimetric assay previously reported [45]. The result is given as an IC_50_ value in µg/mL.

## 4. Conclusions

In his early work on the sterols of marine algae, Patterson reported that cholesterol (**5**) is the major sterol in most of the red algae (Rhodophyta) [20]. This finding was confirmed by the present study on various red algae collected at the coast of Madagascar. Our study represents one of the most extensive investigations of red algae from this region. Cholesterol (**5**) and oxygenated cholesterol derivatives have been identified as the major components of *Grateloupia* sp., *Gracilaria corticata*, *Halymenia* sp., *Metamastophora* sp., and *Spyridia* sp. From the extracts of *Spyridia* sp. and *Calloseris* sp. we obtained 24-methyl-25-homocholesterol (**8**) and we achieved the first full assignment of its ^1^H and ^13^C NMR data. The three known brominated indoles **1**–**3** have been isolated from *Laurencia complanata* along with the sesquiterpene debilone (**4**). For the first time, we have isolated debilone (**4**) from a marine organism. From the extracts of *Calloseris* sp. we achieved the second isolation of the two A-ring contracted steroids (−)-2-ethoxycarbonyl-2β-hydroxy-A-nor-cholest-5-en-4-one (**9**) and phorbasterone B (**10**). A mixture of the three fatty acid esters **12**–**14** with an internal allylic alcohol function has been obtained from *Neurymenia fraxinifolia*. These compounds probably derive from autoxidation of palmitoleates and oleates. We have screened the crude plant extracts for their antimicrobial activity. The extract of *Laurencia* *complanata* showed the highest antimicrobial activities against *Bacillus cereus*, *Staphylococcus aureus*, *Streptococcus pneumoniae*, and *Candida albicans*.

## Supplementary Files

Supplementary File 1
