# Supplementary material for: Red Algae (Rhodophyta) from the Coast of Madagascar: Preliminary Bioactivity Studies and Isolation of Natural Products
Source: Mar Drugs. 2015 Jul 7;13(7):4197–216. doi: 10.3390/md13074197 (PMC4515612; doi:10.3390/md13074197)
Supplement: Supplementary File 1 [file marinedrugs-13-04197-s001.docx]

**Supplementary Information**


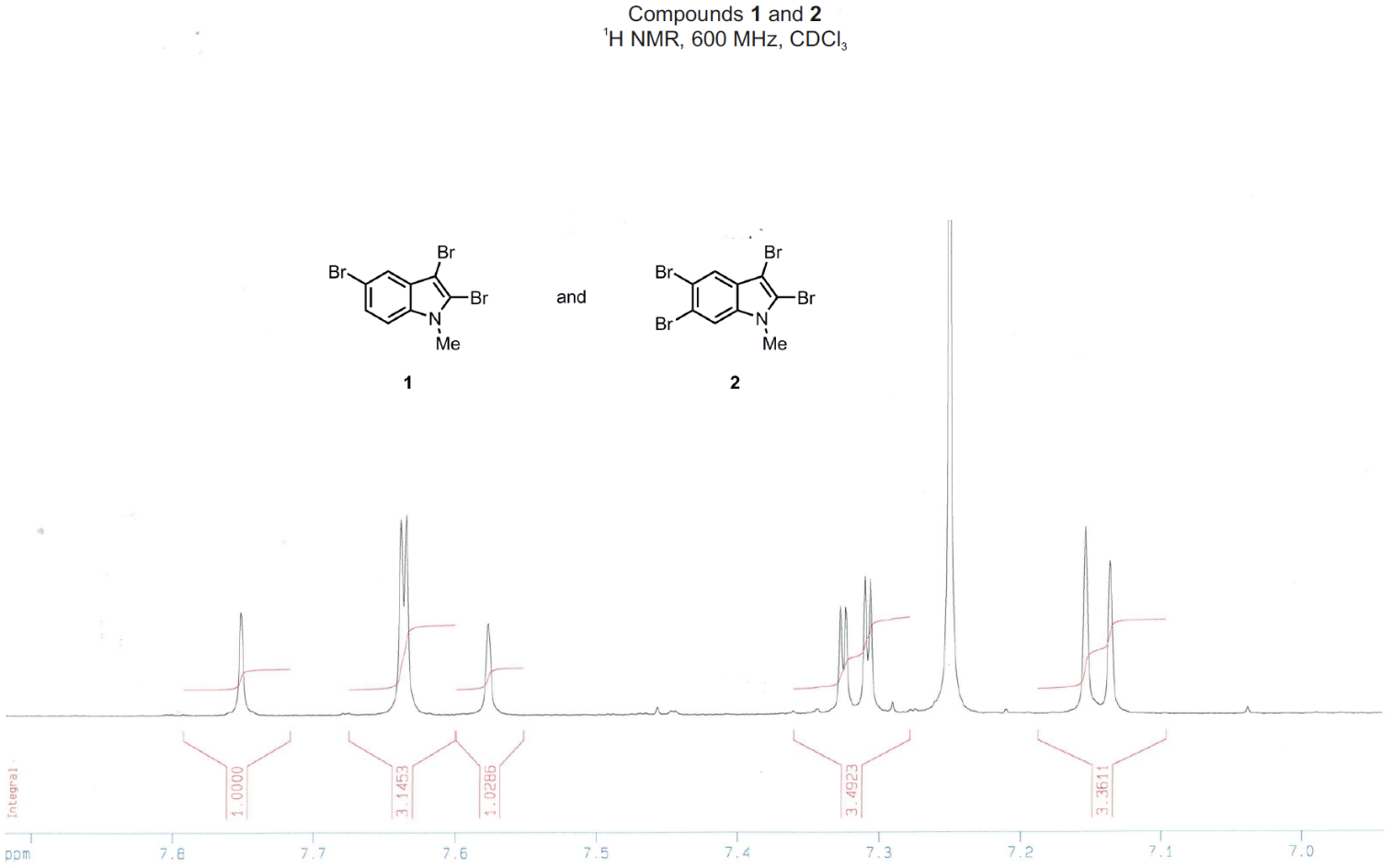


**Figure S1.** ^1^H NMR Spectrum of compounds **1** and **2**.


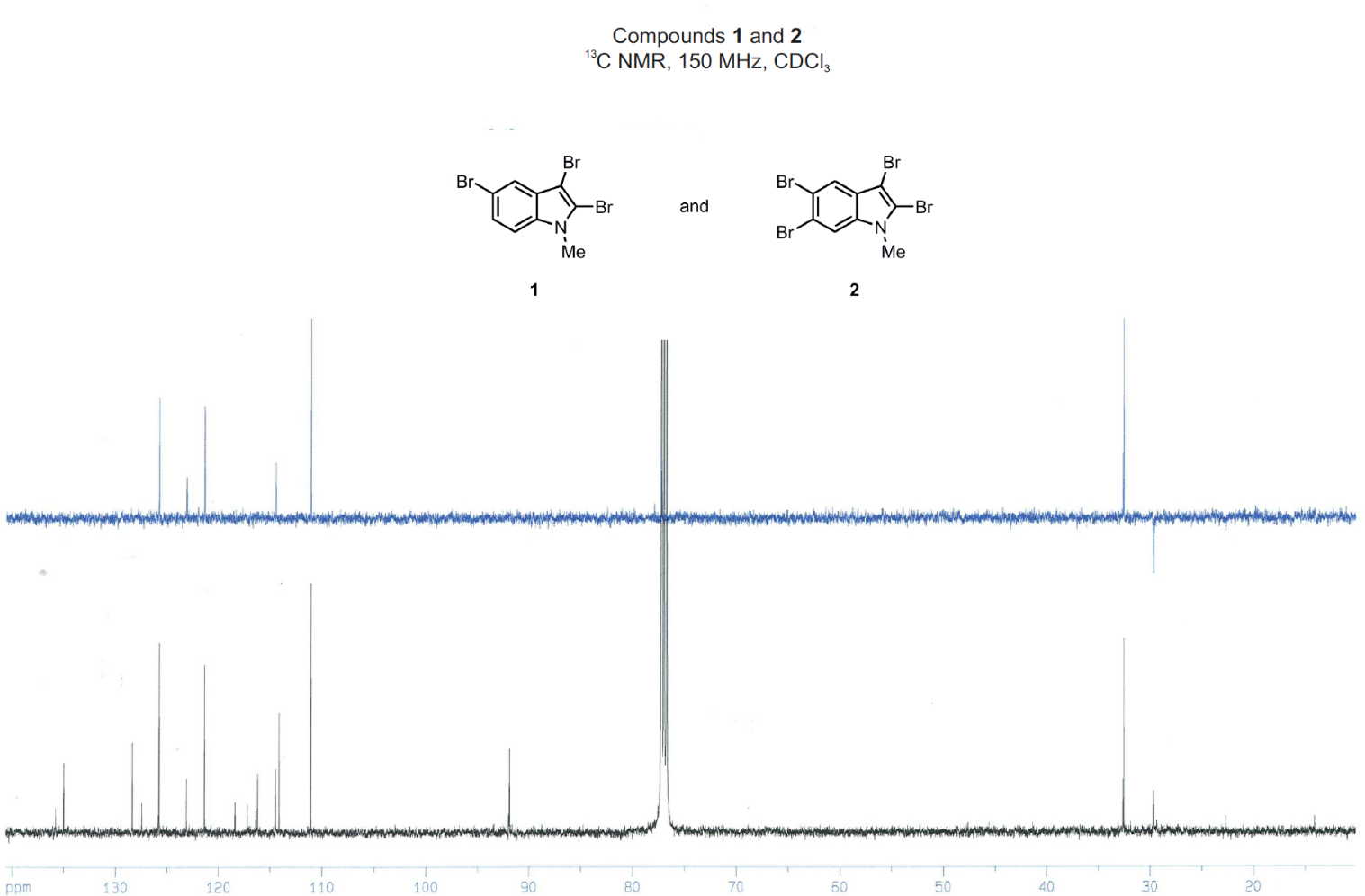


**Figure S2.** ^13^C NMR Spectrum of compounds **1** and **2**.


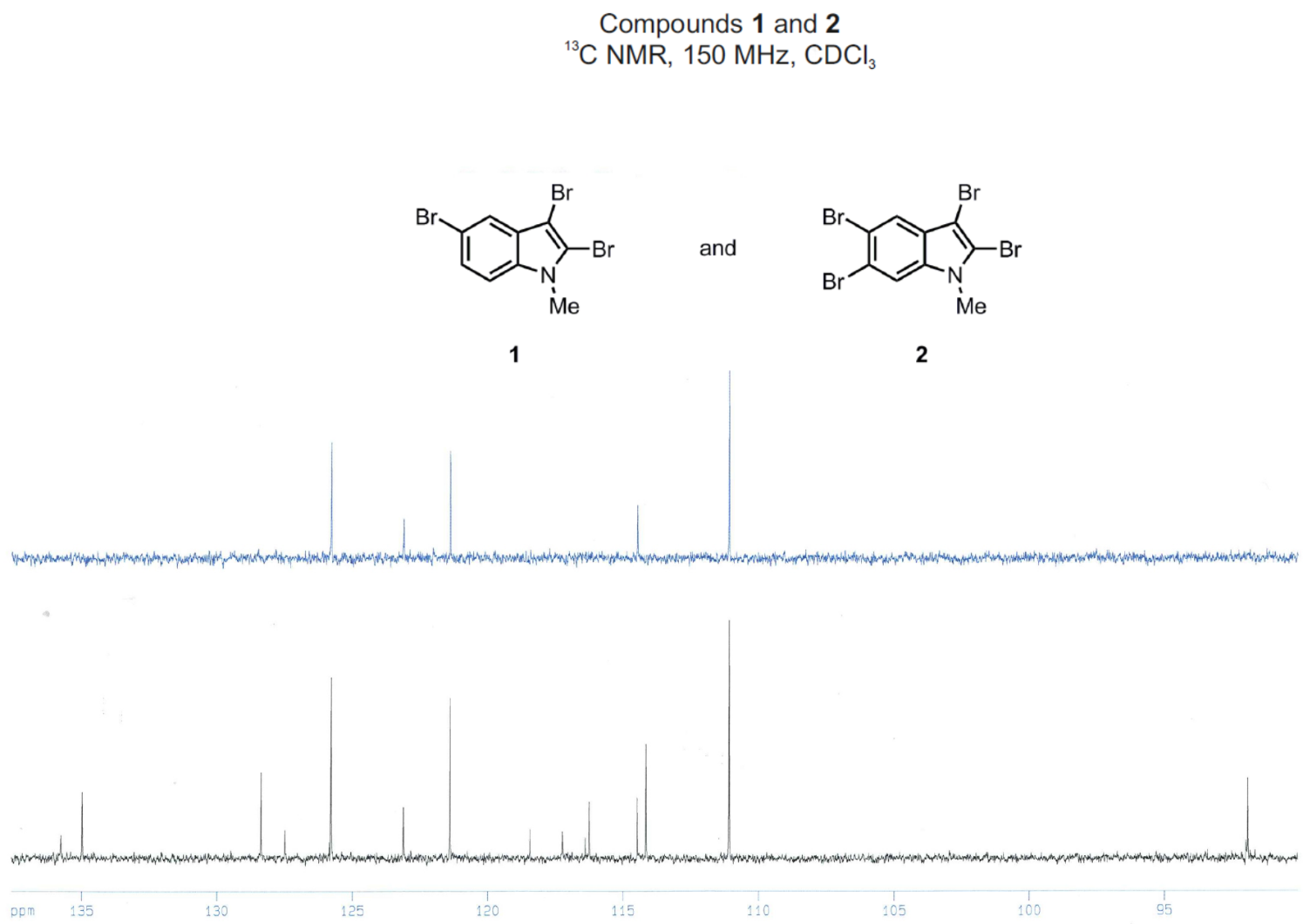


**Figure S3.** ^13^C NMR Spectrum of compound **1** and **2**.


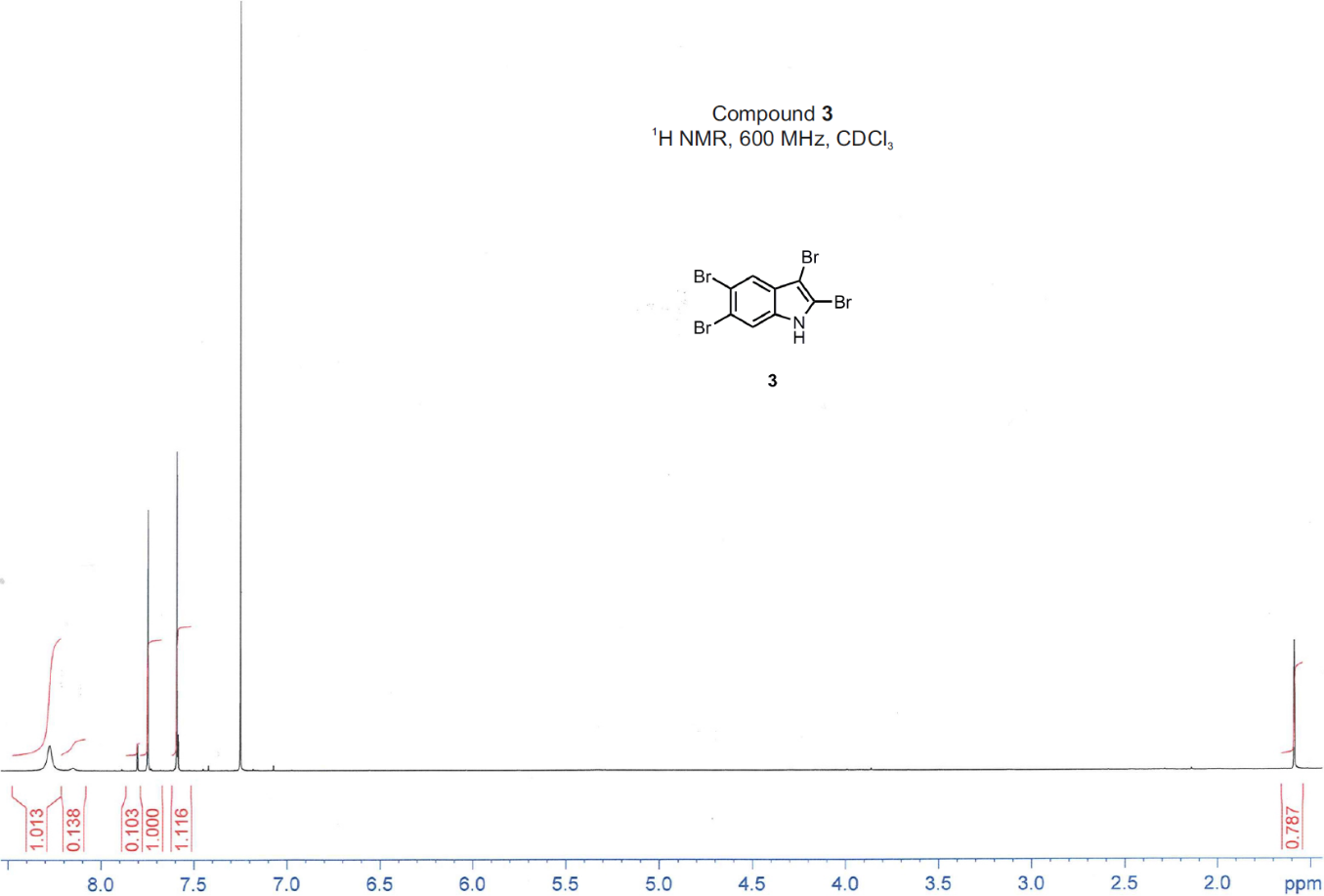


**Figure S4.** ^1^H NMR Spectrum of compound **3**.


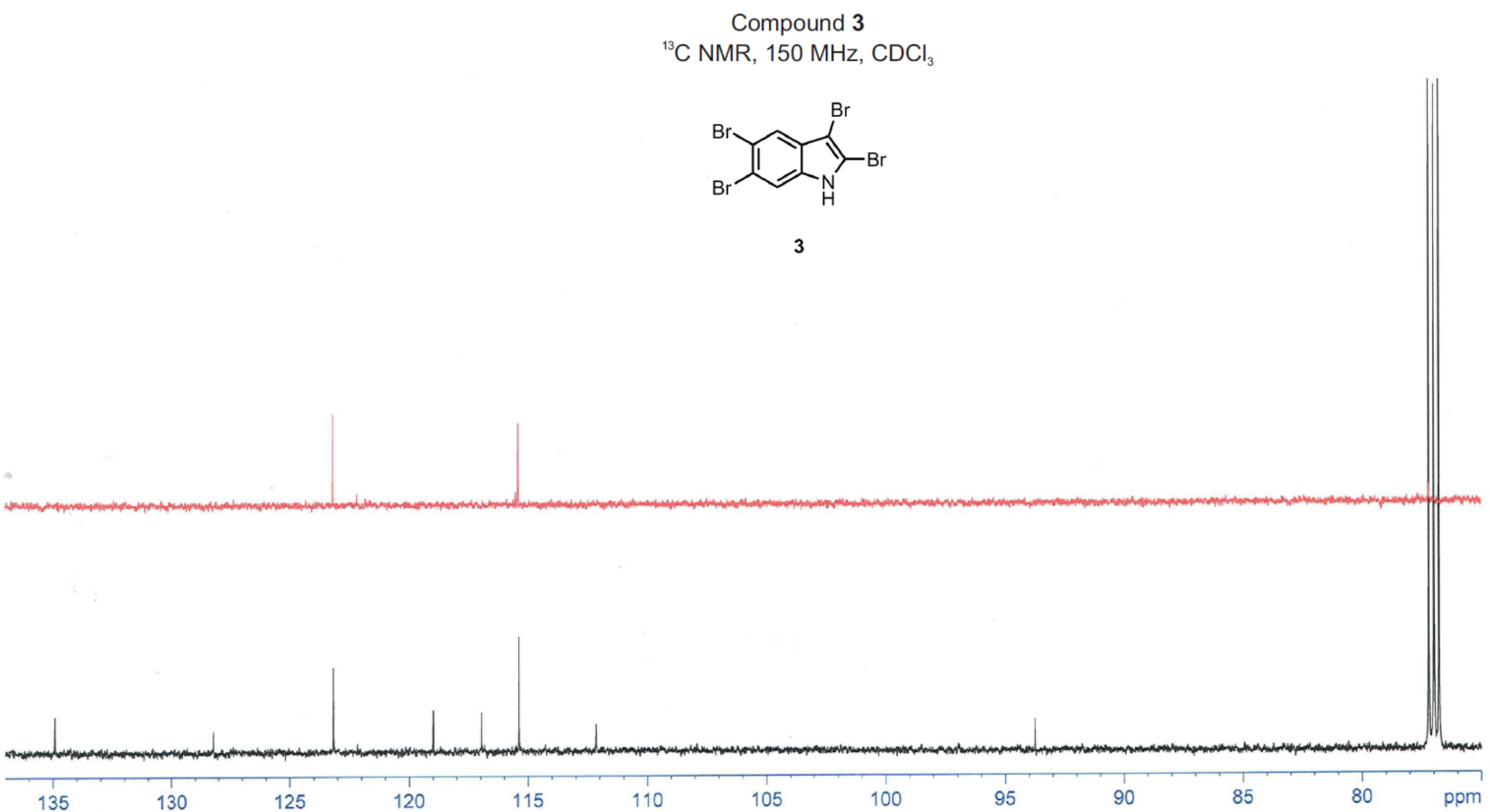


**Figure S5.** ^13^C NMR Spectrum of compound **3**.


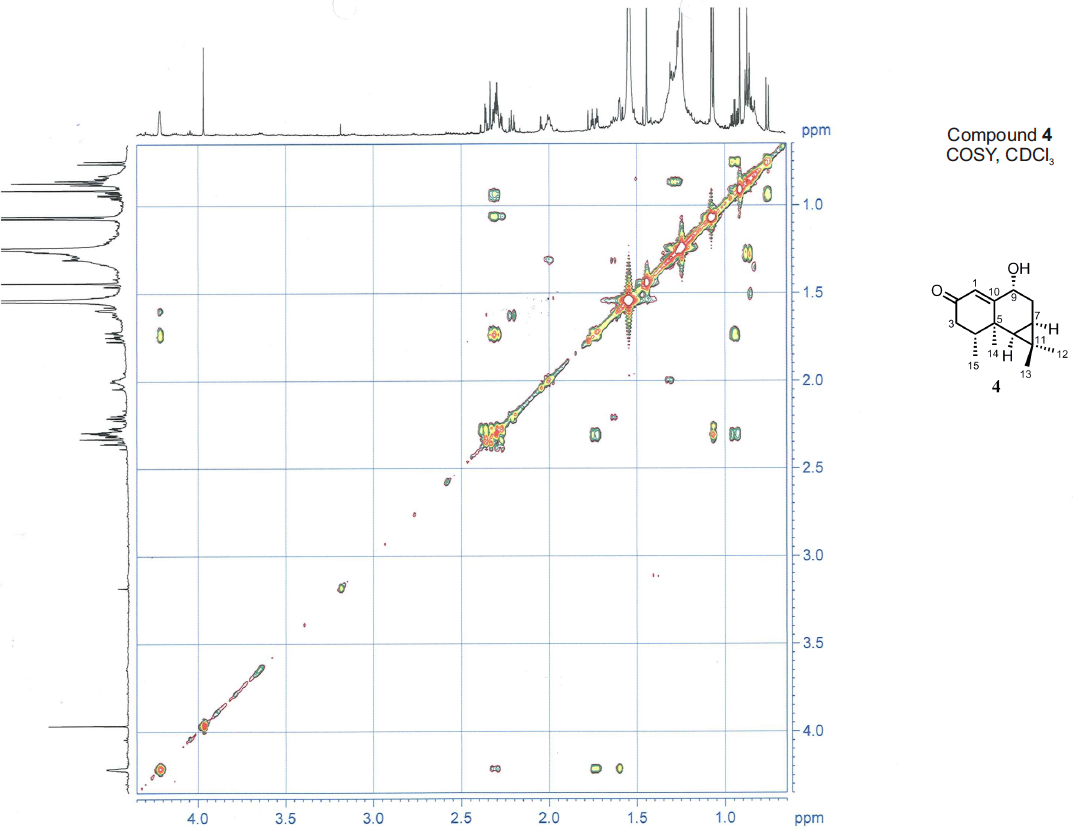


**Figure S6.** COSY Spectrum of compound **4**.

**Figure S7.** COSY Spectrum of compound **4**.

**Figure S8.** HSQC Spectrum of compound **4**.


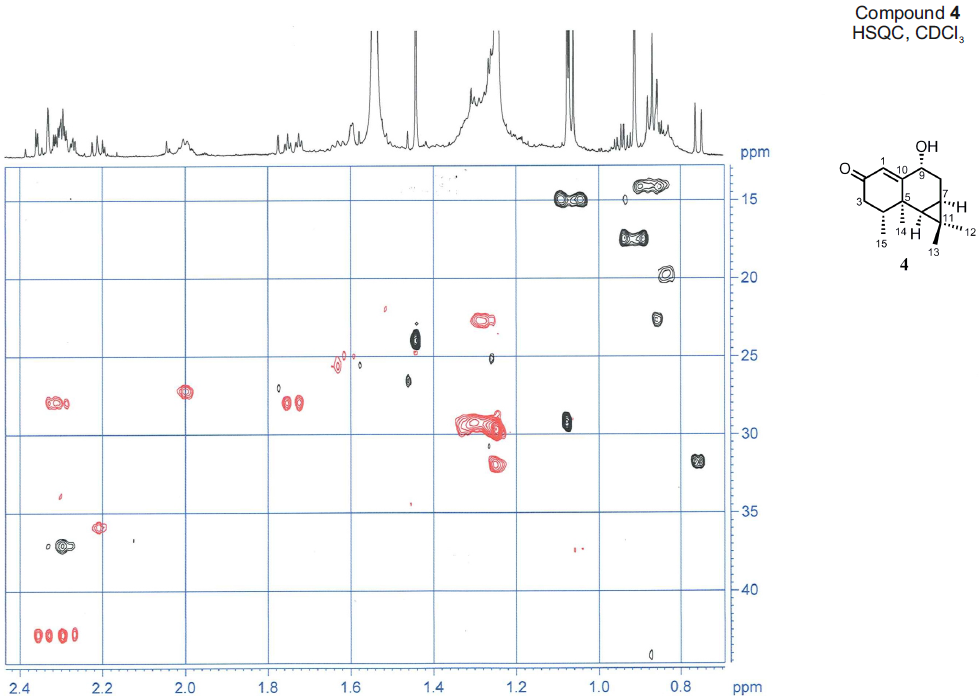


**Figure S9.** HSQC Spectrum of compound **4**.


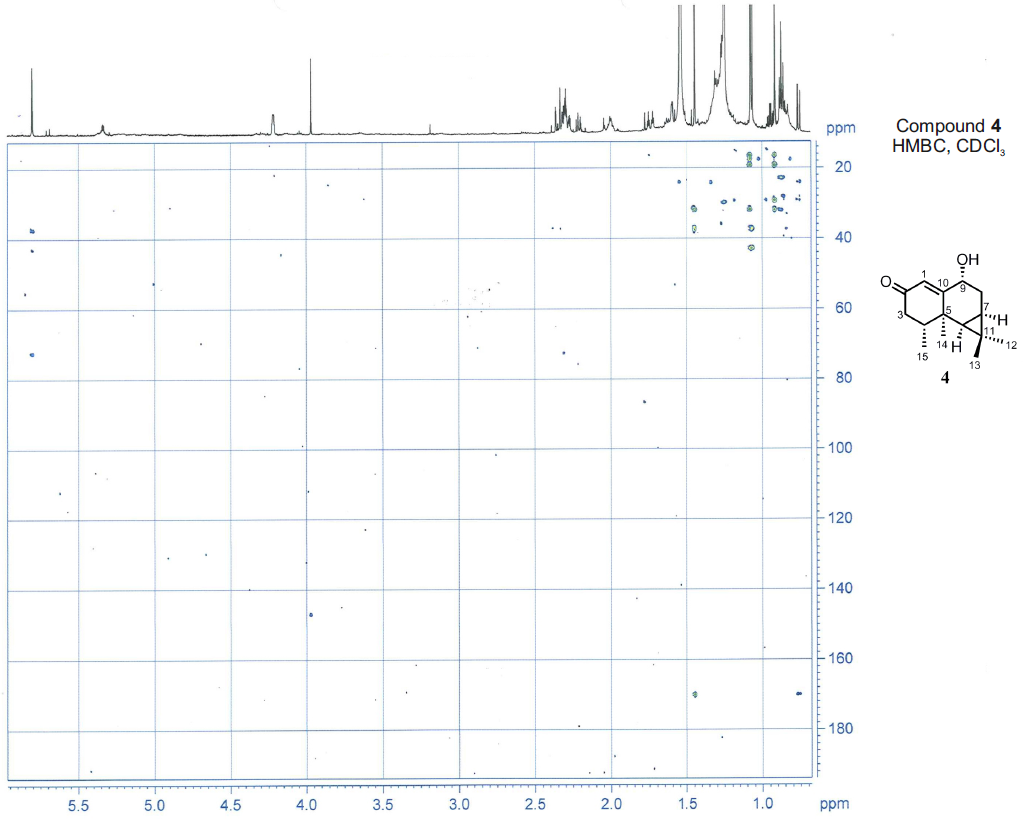


**Figure S10.** HMBC Spectrum of compound **4**.

**Figure S11.** HMBC Spectrum of compound **4**.


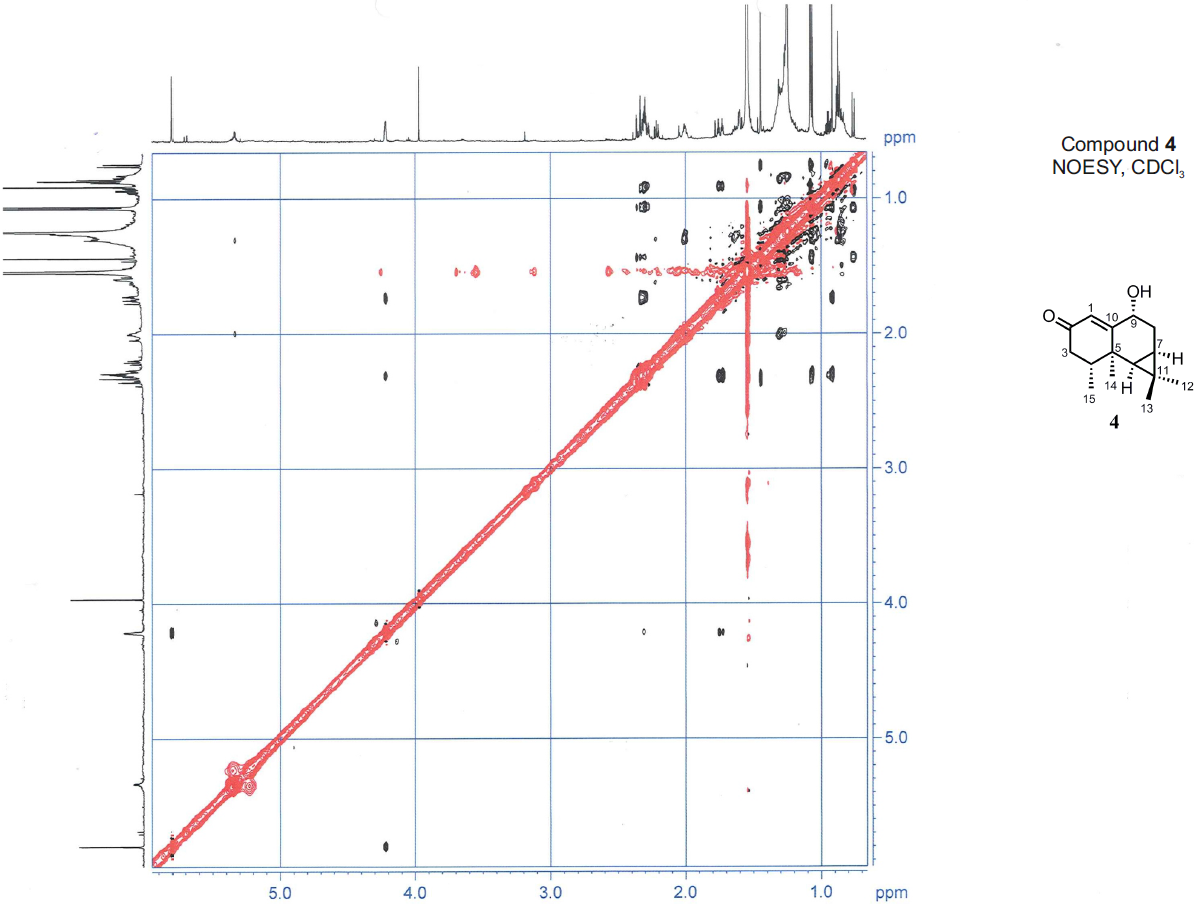


**Figure S12.** NOESY Spectrum of compound **4**.


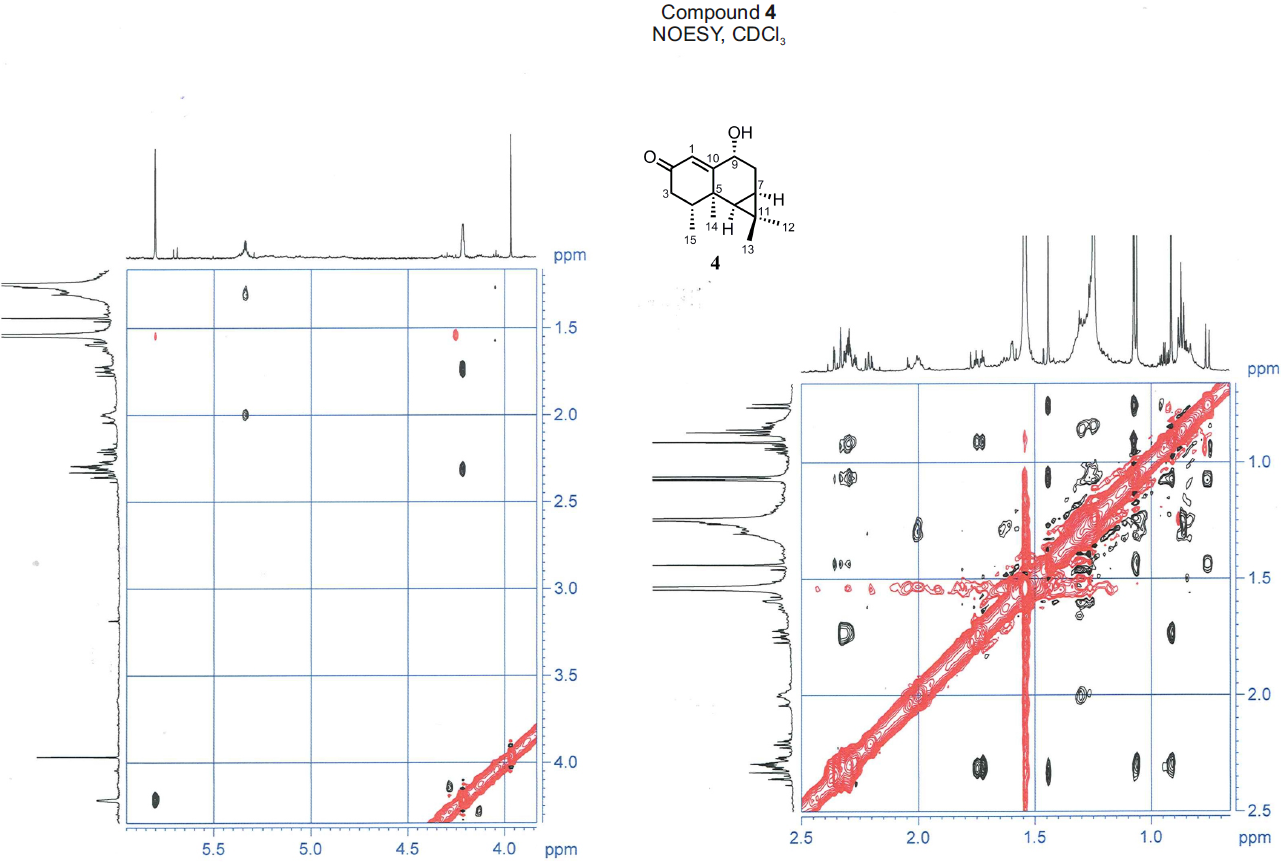


**Figure S13.** NOESY Spectrum of compound **4**.

**Figure S14.** ^1^H NMR Spectrum of compound **8**.


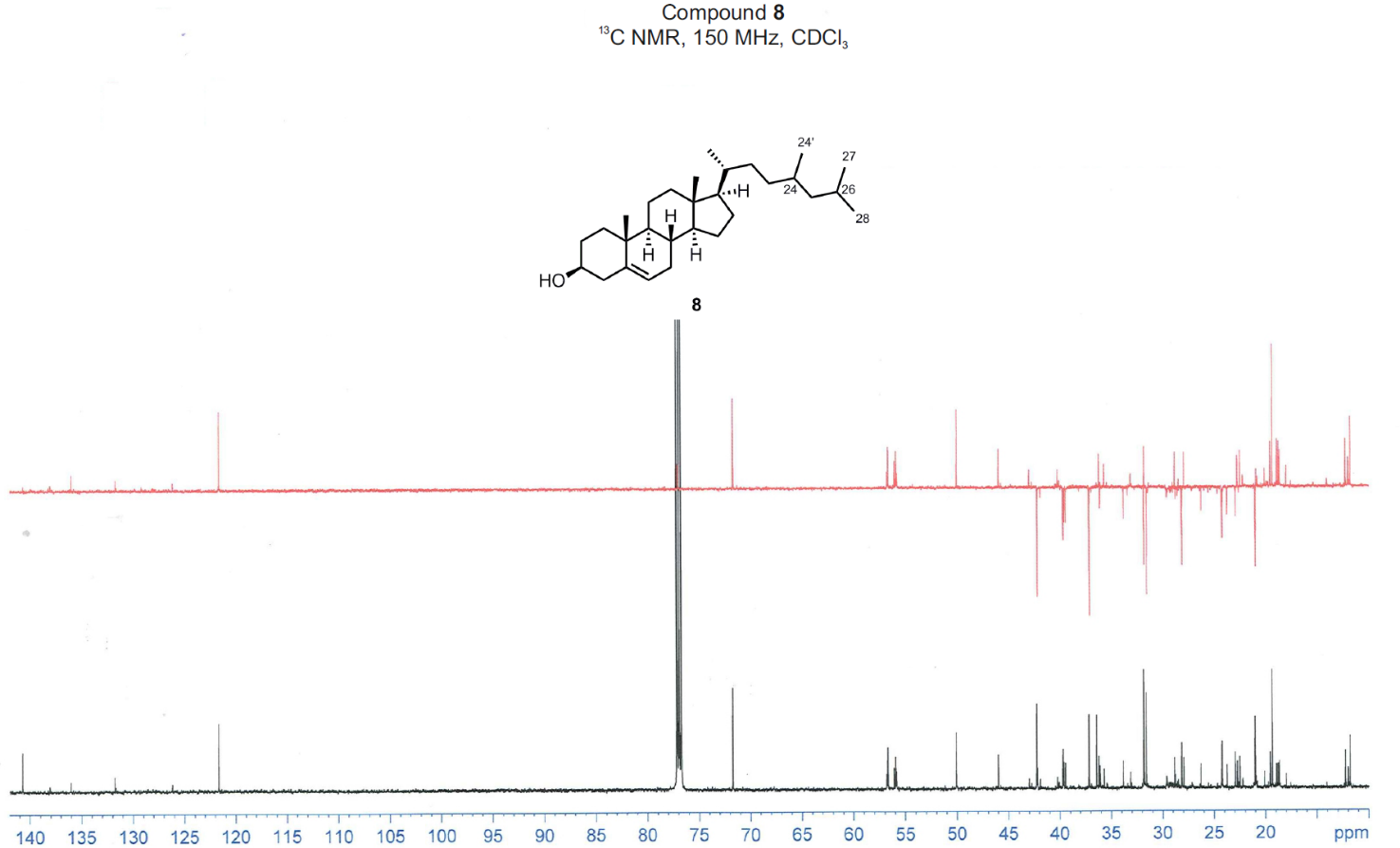


**Figure S15.** ^13^C NMR Spectrum of compound **8**.


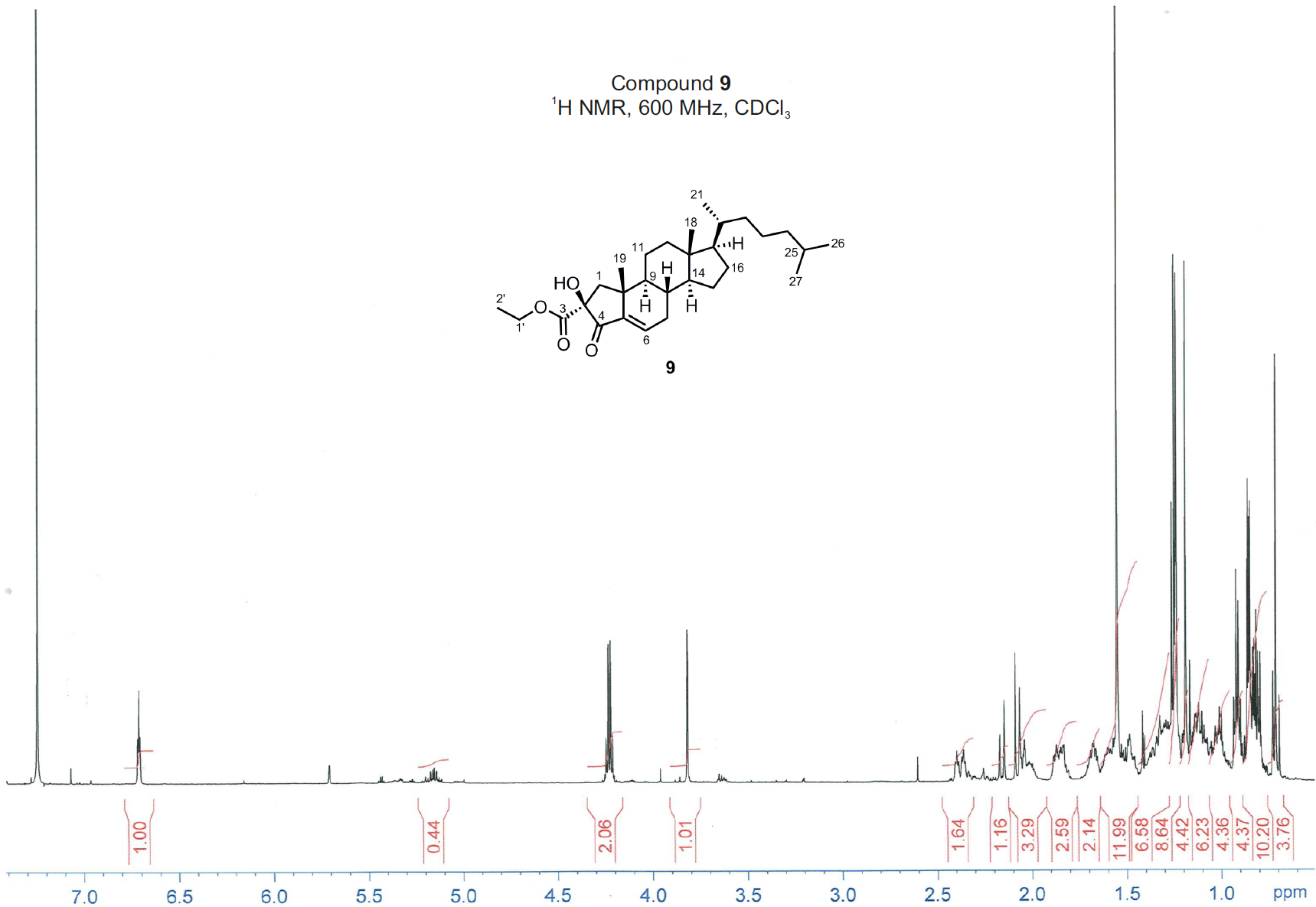


**Figure S16.** ^1^H NMR Spectrum of compound **9**.


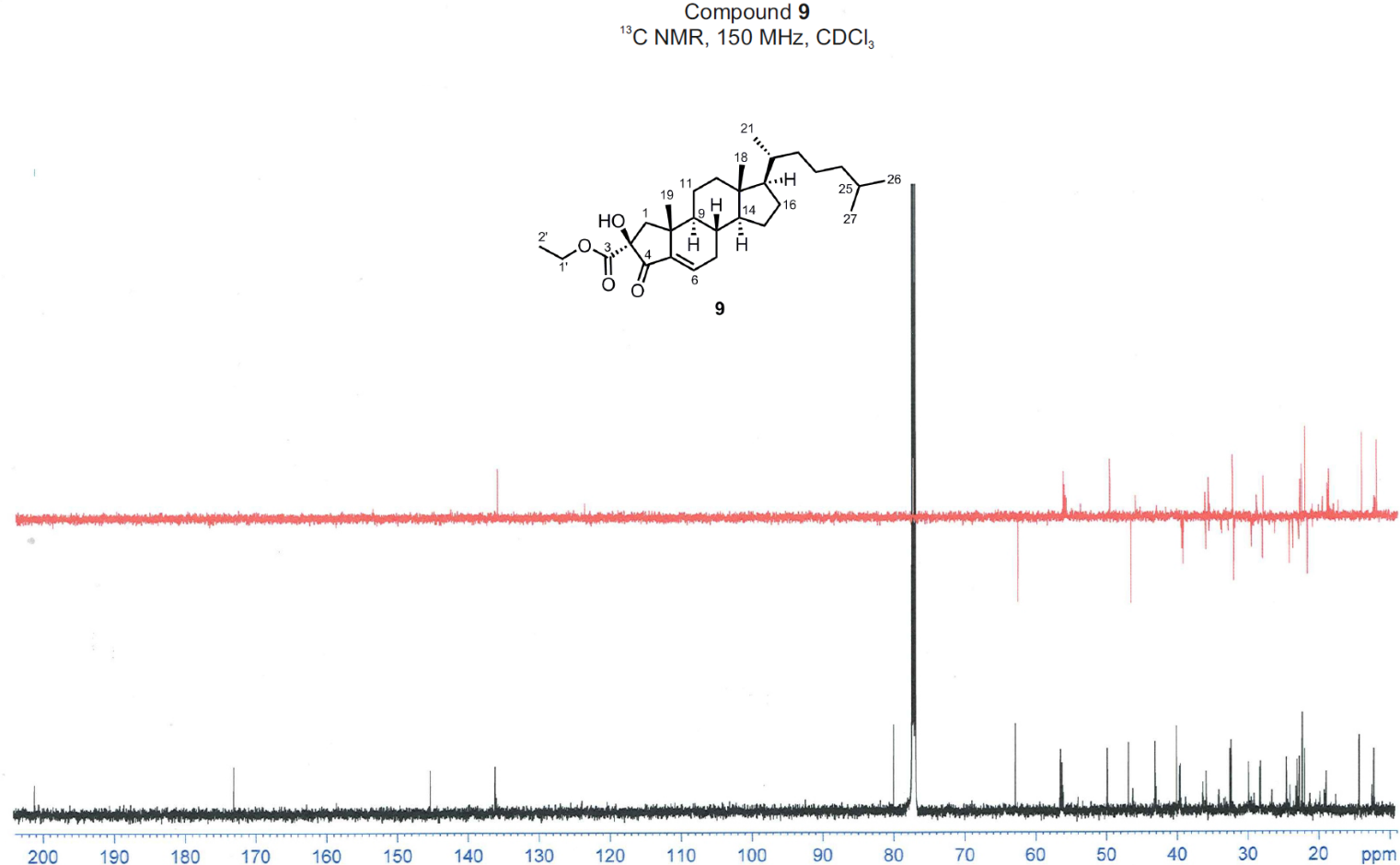


**Figure S17.** ^13^C NMR Spectrum of compound **9**.


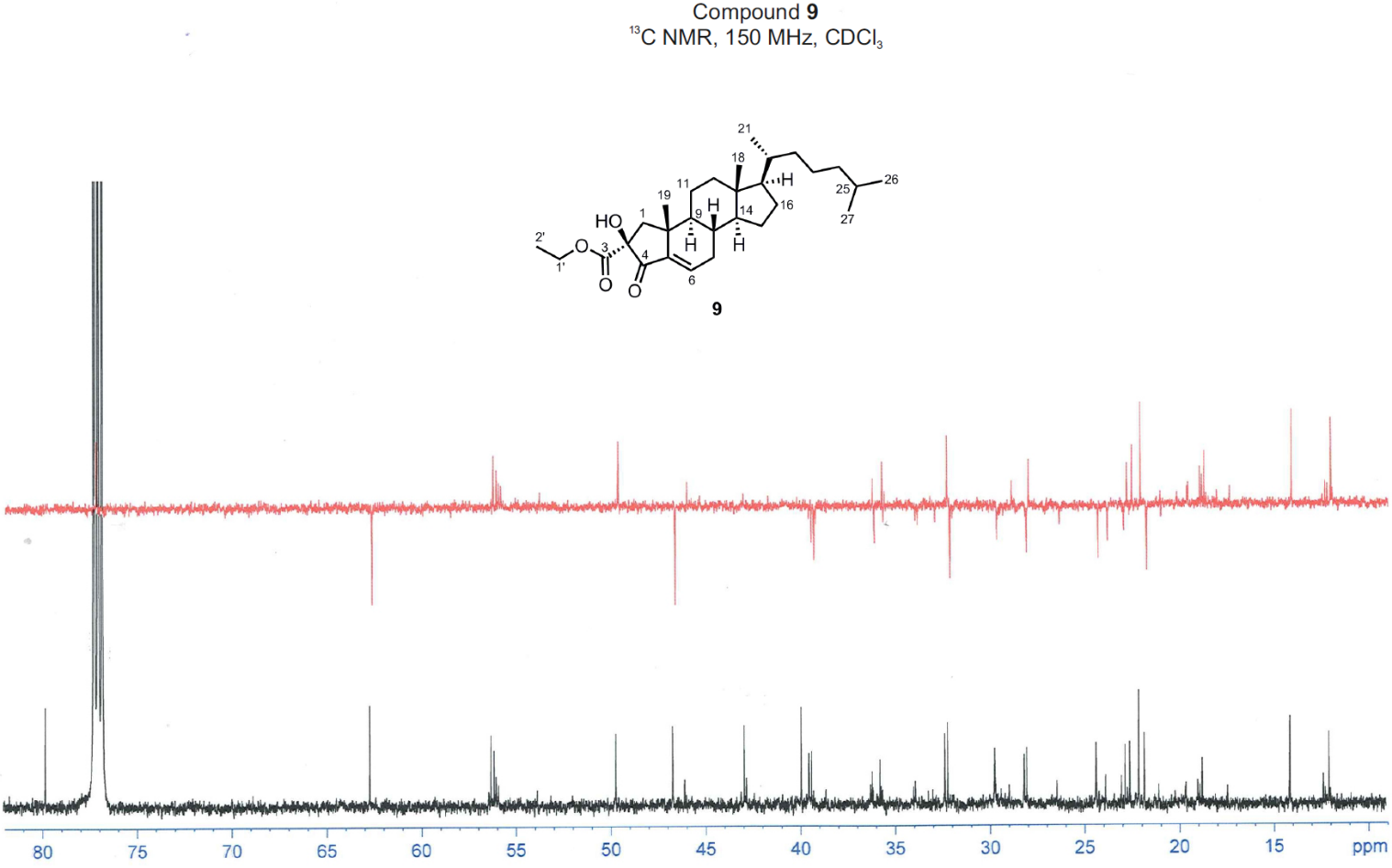


**Figure S19.** ^13^C NMR Spectrum of compound **9**.
